# Unraveling negative biotic interactions determining soil microbial community assembly and functioning

**DOI:** 10.1038/s41396-021-01076-9

**Published:** 2021-07-28

**Authors:** Sana Romdhane, Aymé Spor, Julie Aubert, David Bru, Marie-Christine Breuil, Sara Hallin, Arnaud Mounier, Sarah Ouadah, Myrto Tsiknia, Laurent Philippot

**Affiliations:** 1 Université Bourgogne Franche-Comté, INRAE, AgroSup Dijon, Agroécologie, Dijon, France; 2 Université Paris-Saclay, AgroParisTech, INRAE, UMR MIA-Paris, Paris, France; 3 Department of Forest Mycology and Plant Pathology, Swedish University of Agricultural Sciences, Uppsala, Sweden; 4 Soil Science and Agricultural Chemistry Lab, Department of Natural Resources and Agricultural Engineering, Agricultural University of Athens, Athens, Greece

**Keywords:** Soil microbiology, Ecology

## Abstract

Microbial communities play important roles in all ecosystems and yet a comprehensive understanding of the ecological processes governing the assembly of these communities is missing. To address the role of biotic interactions between microorganisms in assembly and for functioning of the soil microbiota, we used a top-down manipulation approach based on the removal of various populations in a natural soil microbial community. We hypothesized that removal of certain microbial groups will strongly affect the relative fitness of many others, therefore unraveling the contribution of biotic interactions in shaping the soil microbiome. Here we show that 39% of the dominant bacterial taxa across treatments were subjected to competitive interactions during soil recolonization, highlighting the importance of biotic interactions in the assembly of microbial communities in soil. Moreover, our approach allowed the identification of microbial community assembly rule as exemplified by the competitive exclusion between members of Bacillales and Proteobacteriales. Modified biotic interactions resulted in greater changes in activities related to N- than to C-cycling. Our approach can provide a new and promising avenue to study microbial interactions in complex ecosystems as well as the links between microbial community composition and ecosystem function.

## Introduction

Microbial communities in nature exist in complex and dynamic consortia of populations that are not only central to all major biogeochemical cycles, but also influence plant, animal, and human welfare [1–3]. These communities assemble through neutral processes, as well as through abiotic and biotic filtering [4]. While a large body of research has focused on the importance of abiotic factors [5, 6], there have been relatively less investigations on the importance of biotic factors, and in particular on the interactions between microorganisms, to explain the composition of microbial communities in the environment [7]. Understanding the different processes involved in the assembly of such complex communities is currently receiving attention due to the great potential of translating such knowledge into practical outcomes, e.g., in agroecosystems to increase soil fertility and improve crop production [8, 9].

Various types of positive and negative interactions between microorganisms, ranging from mutualism to competition, have been identified [10–12]. For example, in cooperative interactions, microorganisms can divide labor, whereby some individuals specialize to carry out tasks that benefit other individuals [13]. On the other hand, competition can be fierce between microorganisms, with evidence of both indirect exploitative competition, in which an individual consumes the resources required by another member, and direct interference competition, in which an individual inhibits the growth of another through the synthesis of harmful products [12, 14, 15]. Negative interactions between microorganisms also comprise parasitism and predation with diverse predatory viruses, protists, and even bacteria described from a variety of environments [16–19].

To date, efforts to experimentally identify biotic interactions between microorganisms have typically relied on bottom-up approaches based on synthetic-assemblage experiments conducted in vitro with culturable strains [20–22]. These co-culture experiments are based on the assumption that if there is an interaction between two microbial species, the fitness of at least one of them is different when grown together than when grown in the absence of the other species [14]. Such approaches have provided insights into underlying mechanisms by which microorganisms interact, but do not reflect the complexity of natural microbial communities or of their natural habitat. There is therefore little empirical data regarding the extent to which biotic interactions are shaping the composition of complex microbial communities in natural settings.

Here, we take an alternative top-down approach based on microbial community manipulation by targeted removal of various microbial groups in a native soil community to test the role of biotic interactions for microbial community assembly. Specifically, soil microbial communities were first subjected to different biocidal and filtration treatments before being reinoculated in their native, but sterilized soil to allow them to assemble during recolonization. Such removal treatments are intuitively predicted to cause changes in the fitness of the microorganisms interacting with those being depleted by the treatment. We hypothesized that manipulation of the microbial community will lead to changes in the community assembly during the soil colonization process in ways that can unravel the importance of biotic interactions, and their consequences for soil functions.

We show here that 39% of the dominant bacterial taxa across treatments were subjected to competitive interactions during soil recolonization, therefore experimentally showing for the first time the importance of negative interactions between microorganisms for community assembly in a complex environment. This removal approach can also provide a new framework to study microbial interactions in ecosystems as well as links between microbial community composition and ecosystem function based on the analogy to gene-knockout procedures in genomics.

## Materials and methods

### Soil sampling and experimental design

**Fig. 1:**
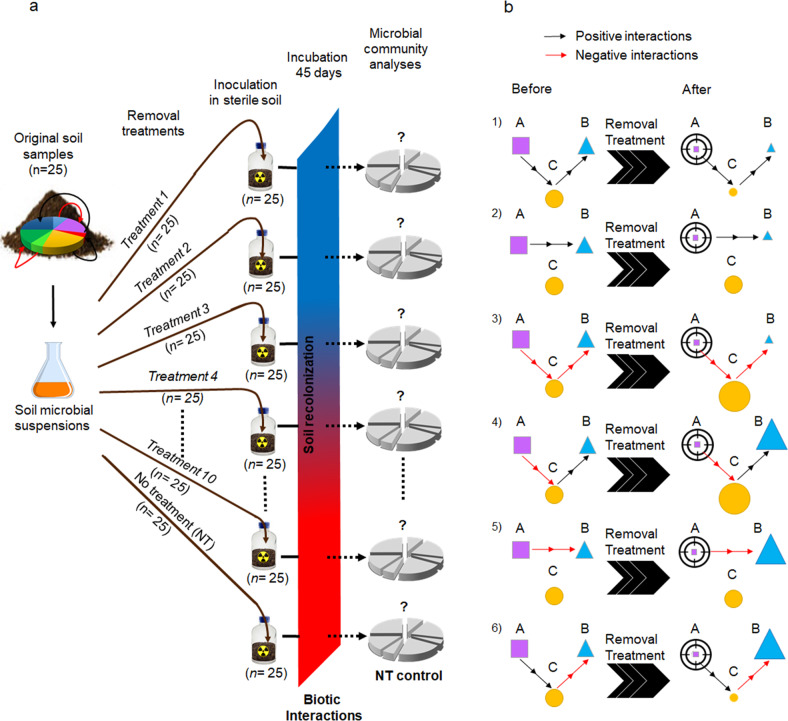
Schematic illustration of the experimental design. **a** Design of the microcosm experiment to manipulate the soil microbial community by subjecting soil suspensions to a range of removal treatments (three antibiotics, one antimicrobial peptide, four size filtration, one heat shock, and one oxidative stress), targeting various microbial groups (*n* = 25). Pie charts are symbolizing the composition of microbial communities. Values in parenthesis indicate the number of replicates. **b** Summary of possible ecological interactions between different species (A, B, and C) in the community (before) and consequences in each case for the other two species (increased or decreased relative fitness represented by the size of the symbols) when species A is depleted by a removal treatment (after). In examples 1–3, depletion of species A caused a decrease in relative fitness of species B, which it is not possible to distinguish from a decrease in relative fitness caused by a direct effect of the removal treatment. Only examples 4–6 causing an increase in the relative fitness are detected in our approach.

The soil was collected from the Epoisses site in France (47° 30′ 22.1832′′ N, 4° 10′ 26.4648′′ E) in March 2017. The soil properties were 41.9% clay, 51.9% silt, and 6.2% sand, pH 7.2, and C and N content 15.5 and 1.4 g kg^−1^ dry soil, respectively. Twenty-five soil subsamples were kept at −20 °C to characterize the initial microbial species pool. The collected soil was sieved through 4 mm before preparing soil suspensions by mixing 100 g equivalent dry mass soil with 150 ml sterile distilled water with a waring blender under sterile conditions. Soil suspensions were diluted ten times, centrifuged at 1000 × g for 2 min and supernatants were then filtrated at 10 µm in order to remove larger microbial communities. Soil suspensions were subjected to ten different treatments aiming at removing various microbial groups: three types of biocidal antibiotics (gentamicin, ramoplanin, and ciprofloxacin), an antimicrobial peptide (RW4), four filtration treatments based on cell size (*F* ≥ 3 µm, 0.8 ≤ *F* < 3 µm, 0.4 ≤ *F* < 0.8 µm, and *F* < 0.4 µm), a heat shock (0 °C for 5 min/70 °C for 15 min/0 °C for 5 min), and oxidative stress treatments (H_2_O_2_ at a final concentration of 50 mM). Each treatment was replicated on 25 soil suspensions and 4.5 mL from each treated soil suspensions was inoculated into 147 mL plasma flasks containing 30 g of the same gamma-sterilized soil (two times 35 kGy; Conservatome, Dagneux, France). All of the 275 soil microcosms were closed with sterile lids and incubated at 20 °C at a soil moisture ranging between 60 and 80% of the soil water-holding capacity for 45 days. Soil microcosms inoculated with non-treated soil suspensions and incubated 45 days (NT control) were used as controls (*n* = 25; Fig. 1).

### Assessment of microbial community composition and diversity

After 45 days of incubation, soil microcosms were used for analyses of total bacterial and fungal diversity and composition by sequencing the 16S rRNA and ITS genes via Illumina Miseq 2 × 250 bp paired-end analysis. First, DNA was extracted from 250 mg from each of the 275 soil microcosms as well as the 25 original soil subsamples using the DNeasy PowerSoil-htp 96-well DNA isolation kit (Qiagen, Hilden, Germany). Amplicons were generated for all 300 DNA extracts in two steps. In the first step, the V3-V4 hypervariable region of the bacterial 16S rRNA gene was amplified by polymerase chain reaction (PCR) using the fusion primers U341F (5’-CCTACGGGRSGCAGCAG-3’) and 805R (5’-GACTACCAGGGTATCTAAT-3’), with overhang adapters (forward: TCGTCGGCAGCGTCAGATGTGTATAAGAGACAG, adapter: GTCTCGTGGGCTCGGAGATGTGTATAAGAGACAG) to allow the subsequent addition of multiplexing index-sequences. Fungal ITS was amplified using the primers ITS3F (5’-GCATCGATGAAGAACGCAGC-3’) and ITS4R (5’-TCCTCSSCTTATTGATATGC-3’). Thermal cycling conditions of the first step PCR were as follows: 98 °C for 3 min followed by 98 °C for 30 s, 55 °C for 30 s, and 72 °C for 30 s (25 and 30 cycles for 16S rRNA and ITS genes, respectively) and a final extension for 10 min at 72 °C. Duplicate first step PCR products were pooled and then used as template for the second step PCR. In the second step, PCR amplification added multiplexing index-sequences to the overhang adapters using a unique multiplex primer pair combination for each sample. Thermal cycling conditions were as follows: 8 °C for 3 min followed by 98 °C for 30 s, 55 °C for 30 s, and 72 °C for 30 s (8 and 10 cycles for 16S rRNA and ITS genes, respectively) and a final extension for 10 min at 72 °C. Duplicate second step PCR products were pooled then visualized in 2% agarose gel to verify amplification and size of amplicons. The amplicons were cleaned-up and pooled using sequalPrep Normalization plate kit 96-well (Invitrogen, Carlsbad, CA, USA). Sequencing was performed on MiSeq (Illumina, 2 × 250 bp) using the MiSeq reagent kit v2 (500 cycles). Demultiplexing and trimming of Illumina adaptors and barcodes was done with Illumina MiSeq Reporter software (version 2.5.1.3).

### Sequencing and bioinformatic analysis

Sequence data from the 300 soil samples were analyzed using an in-house developed Python pipeline (available upon request). Briefly, 16S rDNA and ITS sequences were assembled using PEAR [23] with default settings. Further quality checks were conducted using the QIIME pipeline [24] and short sequences were removed (<400 bp for 16S and <300 bp for ITS). Reference-based and de novo chimera detection, as well as operational taxonomic units (OTUs) clustering were performed using VSEARCH [25] and the adequate reference databases (SILVA representative set of sequences for 16S rRNA and UNITE’s ITS2 reference dynamic dataset for ITS). The identity thresholds were set at 94% for 16S rRNA based on replicate sequencing of a bacterial mock community [26] and 97% for ITS. A total of 4,307,710 bacterial 16S rRNA gene and 15,077,367 fungal ITS region sequences were obtained. Representative sequences for each OTU were aligned using PyNAST [27] and a 16S rDNA phylogenetic tree was constructed using FastTree [28]. Taxonomy was assigned using UCLUST [29] and the SILVA reference database 132 [30]. For ITS, the taxonomy assignment was performed using BLAST [31] and the UNITE reference database (v.7-08/2016 [32]). Raw sequences were deposited at the NCBI under the BioProject PRJNA542862.

Bacterial and fungal α-diversity metrics (i.e., observed species, Simpson’s reciprocal, Shannon, and for bacteria also Faith’s Phylogenetic Diversity PD [33]) and Net Relatedness and Nearest Taxon indices [34] were calculated based on rarefied OTU tables (5000 sequences per sample for 16S rDNA and 8000 sequences per sample for ITS). To assess the contribution of deterministic and stochastic processes to the bacterial community structure, the normalized stochasticity ratio index (NST) was calculated [35]. Weighted UniFrac distance matrix [36] and Bray–Curtis dissimilarity matrix were also computed to detect variations in the structure of microbial communities for 16S rDNA and ITS, respectively.

Low-abundance OTUs were discarded by keeping OTUs with at least 0.5% relative abundance across all samples (353 and 1370 OTUs for 16S rDNA and ITS, respectively). Due to the high proportion of zero counts, fungal OTUs with low prevalence (present in less than 70% of replicates per treatment) were removed (305 OTUs).

### Quantification of microbial communities

The abundances of total bacterial and fungal microbial communities as well as that of N-cycle microbial guilds were estimated by real-time quantitative PCR (qPCR) assays. For each treatment, the 25 DNA extracts were used to prepare 5 equimolar DNA mixtures (each corresponding to five different DNA extracts), which were added as templates for the qPCR assays (*n* = 5). Total bacterial and fungal communities were quantified using 16S rDNA and ITS primers described by Muyzer et al. [37] and White et al. [38], respectively. The nitrification gene *amoA* and the denitrification genes *nirK* and *nirS* were used as molecular markers to quantify the bacterial (AOB) and archaeal (AOA) ammonia-oxidizing and the denitrifying communities, as described previously [39]. qPCR reactions were carried out in a ViiA7 (Life Technologies, Carlsbad, CA, USA) in a 15 µL reaction volume containing 7.5 µL of Takyon Master Mix (Eurogentec, Liège, Belgium), 1 µM of each primer, 250 ng of T4 gene 32 (MP Biomedicals, Santa Ana, CA, USA), and 1 ng of DNA. Two independent runs were performed for each real-time PCR assay. Standard curves were obtained using serial dilutions of linearized plasmids containing appropriated cloned targeted genes from bacterial strains or environmental clones. PCR efficiency for the different assays ranged from 77 to 101%. No-template controls gave null or negligible values. Inhibition in qPCR assay was tested by mixing soil DNA extracts with either control plasmid DNA (pGEM-T Easy Vector, Promega, Madison, WI, USA) or water. No inhibition was detected in any case.

### Assessing soil functions related to carbon and nitrogen cycling

Causal effects of microbial community manipulations on soil functioning were assessed by measuring a range of activities related to C and N cycles in replicate soil samples from each treatment (*n* = 5). The MicroResp method was used to measure microbial respiration rates of different C substrates across different treatments as described by Campbell et al. [40]. Eleven substrates were used: D-(+)-galactose, L-Arginine, Citric acid, L-Alanine, L-Malic acid, L-(+)-Arabinose, N-Acetyl glucosamine, Glucose phosphate, D-(-)-Fructose, D-(+)-Trehalose, and Gallic_acid. Soil nitrogen pools (NO_3_^–^ and NH_4_^+^) were extracted using 50 mL of 1 M KCl that was added to ca. 10 g fresh soil, shaken vigorously (80 rpm for 1 h at room temperature), filtered and kept frozen until quantification according to ISO standard 14256-2. Quantification was performed by colorimetry in a BPC global 240 photometer.

### Statistical analyses

Statistical analyses were conducted using R statistical software version 3.4.1 [41]. Differences between treatments in gene copy abundances (16S rRNA, ITS, bacterial and archaeal *amoA*, *nirK*, and *nirS*), ammonium and nitrate concentrations, microbial respiration measurements (*n* = 5) and the microbial α-diversity indices (*n* = 25) were tested using ANOVAs followed by Tukey’s honestly significant difference test (*p* value < 0.05) using the agricolae package [42]. Normality and homogeneity of the residual distribution were verified and log-transformations were performed when necessary.

#### Evaluating the impact of the removal treatments on beta-diversity

Permutational multivariate analysis of variance (PermANOVA) was carried out on the weighted UniFrac and the Bray–Curtis dissimilarity distance matrices to detect significant differences between treatments (*n* = 25) in community composition using adonis function implemented in the vegan package [43]. Principal coordinates analysis (PCoA) visualizations were created using the plot3D package using scatter3D function [44].

#### Identification of OTUs significantly affected by the removal treatments

Differential abundance analysis of microbial community composition was performed by comparing the count matrices between each treatment (*n* = 25) and the NT control (*n* = 25) using the default parameters of DESeq2 Bioconductor package (v.1.30.1), which allows for testing for changes in count matrices between conditions based on negative binomial generalized linear models [45, 46]. Significant OTUs result from Benjamini–Hochberg adjusted *p* values (BH-adjusted *p* value < 0.00001). Because of the high variability in the distribution of fungal sequences across replicates, only OTUs having a coefficient of variation lower than 200% within a given treatment and within NT control were kept for the DESeq2 analysis.

Bacterial OTUs exhibiting significant changes were used to build pruned trees using the ape package [47] and the trees were visualized using the Interactive Tree of Life [48].

#### Co-occurrence networks construction

Bacterial networks were constructed based on OTU count data (353 OTUs) using both all treatments plus the NT control for the global network (275 samples). Microbial inter-domain network was constructed using filtered bacterial and fungal OTU tables (353 and 305 OTUs, respectively) from all treatments plus the NT control (275 samples). For the original soil samples (25 samples), raw read count tables of both bacteria and fungi were used after removing OTUs that are not present in all replicates. Networks were inferred using a sparse multivariate Poisson log-normal (PLN) model with a latent Gaussian layer and an observed Poisson layer using the PLNmodels package [49]. The best network was selected using a Stability Approach to Regularization Selection [50]. An inter-domain-specific normalization with the GMPR (geometric mean of pairwise ratios) method was performed to take into account the heterogeneity of sequencing depth [51]. For visualization purpose, only partial correlations with |ρ| > 0.1 were considered. Networks were visualized using the Cytoscape software [52]. Nodes corresponding to the OTUs that showed significant changes in relative abundance based on the DESeq2 analysis were identified using the merge function in R and colored in the network according to log2-fold changes (positive, negative, or both depending on the treatment).

#### Multivariate integration of microbial activities, gene copy abundances, and community composition

Integration and visualization of bacterial OTUs (353), gene copy abundances (16S rRNA, bacterial, and archaeal *amoA*, *nirK*, and *nirS*), and microbial activity measurements were realized using the mixOmics package [53] using DIABLO (Data Integration Analysis for Biomarker discovery using a Latent component method for Omics studies) in order to identify correlated variables between different data sets (Pearson’s correlation |*r*| > 0.7) [54].

## Results and discussion

### Experimental manipulation of microbial community assembly

The experiment takes advantage of the enhanced interactions occurring between microorganisms during their recolonization of sterile soils [55, 56] and we manipulated these interactions using various removal approaches (Fig. 1a). Removal treatments cause depletion of different OTUs, of which some are assumed to have positive or negative interactions with the remaining OTUs. It is therefore expected that the relative fitness of the remaining OTUs that are competing or cooperating with the depleted ones will be modified in the removal treatments during soil recolonization. In this study, it was not possible to distinguish a decrease in relative fitness due to changes in positive interactions from a direct effect of the removal treatment (Fig. 1b). We therefore only focused on negative interactions that caused an increased relative fitness of OTUs that were previously impaired by the depleted ones. Hence, cases of negative interactions for which both competing strains were affected by the removal treatments or causing a decreased relative fitness (example 3, Fig. 1b) could not be determined in our analysis.

**Fig. 2:**
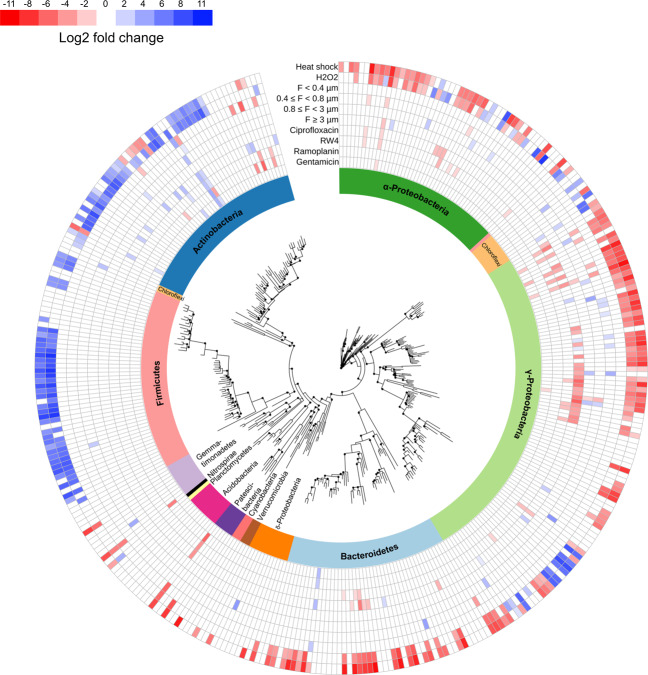
Phylogenetic relationships and distribution of the dominant 353 OTUs. Log2-fold change, as calculated by DESeq2 analysis, of significantly increasing and decreasing bacterial OTUs in the removal treatments when compared to the NT control are represented by the blue-to-red color gradient. The white color indicates OTUs that are not affected by the treatments. The affiliation of OTUs at the phylum or class levels is indicated by different colors on the internal ring. Bootstrap values >80 are indicated by black circles.

A total of 5551 bacterial and 6949 fungal OTUs were found across the different treatments and the original soil samples. To capture the effects of our removal approach on bacterial and fungal communities, differentially abundant OTUs were tested after soil recolonization by pairwise comparisons between the removal treatments and the control without removal treatment (NT control) using DESeq2 [45]. The removal approach effectively led to the depletion of bacterial groups (i.e., with significant decrease in relative abundances in a removal treatment compared to the NT control, BH-adjusted *p* value < 0.00001) that were different between treatments (Fig. 2). For example, we found that members of the Actinomycetes and Bacteroides were affected by the ramoplanin, which is an actinomycete-derived antibiotic. By contrast, ciprofloxacin mostly affected members of the Proteobacteria. Proteobacteria and Bacteroides were also the groups that exhibited the sharpest decline after the oxidative stress and the heat shock treatments. Overall, the depleted OTUs represented between 0.02 and 25% of the total bacterial community in the NT control (and <0.0003–2.3% in the original soil samples in which the five most abundant OTUs represented <5% of the community), which indicates that the selected treatments successfully affected both dominant and rare taxa (Supplementary Fig. 1).

**Fig. 3:**
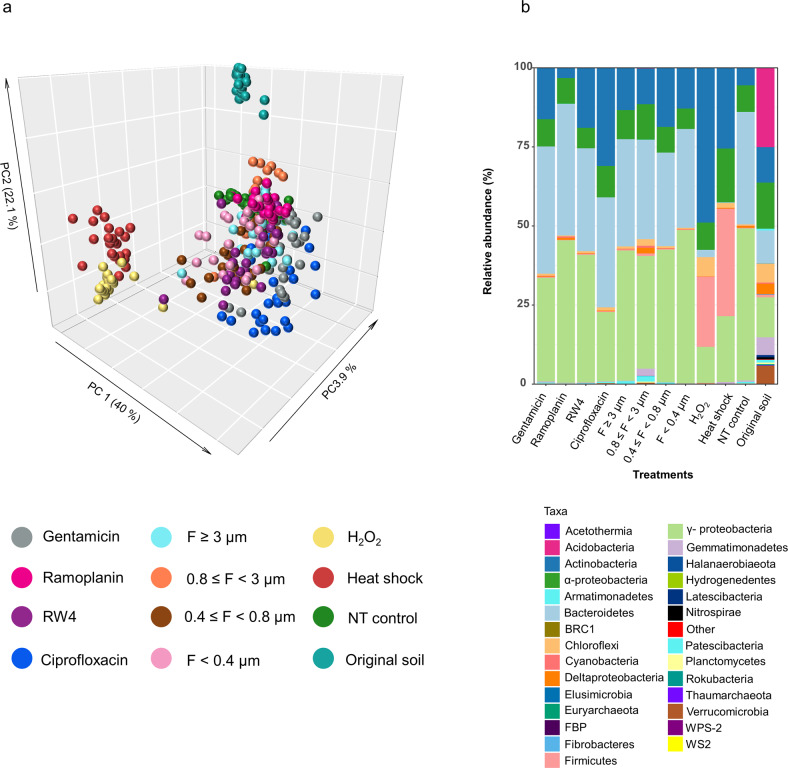
Differences in bacterial community composition across treatments. **a** Principal coordinates analysis (PCoA) of the weighted UniFrac distance matrix of 16S rRNA gene amplicons showing shifts in the bacterial community structures between the original soil, NT control, and removal treatments. The different treatments are represented by different colors as specified in the legend. **b** Bacterial community composition between the original soil samples, NT control, and the different treatments. Relative abundances are shown at the phylum and class levels and expressed as a percentage of the total number of OTUs.

Phylogenetic diversity of OTUs that declined significantly in any of the removal treatments compared to the NT control had a higher degree of relatedness than the total bacterial community (Tukey’s test, *p* value < 0.05; Supplementary Fig. 2). This shows that, as expected, bacterial taxa were mainly non-randomly depleted. UniFrac analyses demonstrated that our manipulation experiment resulted in differences in bacterial community structure between treatments with various degrees of dissimilarity to the NT control (PermANOVA, *p* value < 0.01; Fig. 3a). Major changes were observed in the heat shock and oxidative stress treatments and to a lesser extent in the ciprofloxacin treatments, while the bacterial community structure remained more similar to the control in the ramoplanin and two filtration treatments (Fig. 3 and Supplementary Figs. 3 and 4; Tukey’s test, *p* value < 0.05). The strong clustering by treatment suggests a limited stochasticity during bacterial community assembly despite a few treatments exhibiting more random effects than others (Fig. 3a). This was supported by a NST below 8% for all treatments and NRI/NTI indices higher than 2, which indicates that the coexisting OTUs within treatments were phylogenetically more closely related than expected by chance (Supplementary Figs. 5 and 6). Since the manipulated communities were inoculated in identical soil microcosms, differences in bacterial community assembly were mainly governed by the deterministic effects of the removal treatments in combination with altered species interactions rather than abiotic filtering (i.e., soil properties) or stochastic processes. By contrast, a high variability in the distribution of fungal OTUs was observed between replicates for the manipulated communities, but not in the original soil samples (Supplementary Fig. 7). This was likely due to the breakdown of hyphae during the experimental procedure causing the stochastic distribution of fungal OTUs. We found weak to non-significant effects of the removal treatments on fungal communities with only zero to two fungal OTUs depleted in all treatments but the heat shock treatment, in which nine OTUs mostly belonging to the Hypocreales significantly decreased. Therefore, even if fungi are not excluded, we consider that our experimental approach mainly allows detection of interactions involving bacteria.

Changes in bacterial community composition were concomitant with an increase in bacterial abundance, as determined by qPCR, by at least 1–2 orders of magnitude during soil recolonization. Thus, using the 16S rRNA gene copy number in the original soil to calculate a maximal bacterial density in the inoculum before treatment, we estimated that the inoculation level was less than 10^7^ 16S rRNA gene copies g^−1^ dry soil, while we detected at least 5 × 10^8^ 16S rRNA gene copies g^−1^ dry soil after 45 days (Supplementary Fig. 8). Ecological theory suggests that expansion competition, in which strains race to utilize resources and occupy uninhabited space, is the dominant process determining the outcome of colonization of the sterile soil microcosms by the inoculated communities [57]. Accordingly, the three over-dominant OTUs that were the best at recolonizing the sterile soil in absence of biocidal treatment (NT control) belonged to Bacteroidetes and γ-Proteobacteria, which have previously been reported as copiotrophs (i.e., fast growing in nutrient rich environments) [58–60]. These fast-growing OTUs represented 53% of the total bacterial community in the NT control, but only 2% in the original soil samples despite being among the 25 most dominant OTUs (out of 4057). By contrast, members of the Acidobacteria were common in the original soil, but incapable of thriving in the sterilized soil. Given that soil sterilization by gamma irradiation causes release of organic carbon compounds [61], the disappearance of Acidobacteria supports their proposed oligotrophic lifestyle [58, 59].

### Ecological importance of biotic interactions for microbial community assembly

The successful removal treatments should result in an increased relative abundance of the microorganisms that were subjected to negative interactions with the depleted ones. Due to the compositional nature of the community data, the depletion of certain taxa is also expected to result in an increase in the relative abundance of the other OTUs; with the proportional increase being the same for all the remaining OTUs and corresponding to the fraction of OTUs depleted. However, we observed fold increase in relative abundance that spanned over orders of magnitude for the same removal treatment, which indicates that the relative fitness of some OTUs was stimulated by the depletion of others. Thus, to identify the OTUs showing a significant increase in relative fitness after recolonization compared to the control NT (i.e., higher increase than what would be expected based on just the fraction of OTUs depleted following the removal treatment), a differential abundance analysis of microbial community composition was performed with DESeq2 (negative binomial generalized linear model, BH-adjusted *p* value < 0.00001) (Fig. 1b). Although differential abundance analysis can result in the identification of false positives, DESeq2 has been shown to be conservative and to control well the false positive rate [45]. Furthermore, having 25 replicates per treatment as well as including OTUs with at least 0.5% relative abundance across all samples and removing fungal OTUs with low prevalence, and thereby reducing zero counts, also helped minimizing the number of false positives. Only four fungal OTUs, all belonging to the genus Trichoderma, showed a significantly increased relative fitness across the heat shock and oxidative stress treatments. In contrast to fungi, we found a greater number of negative interactions during bacterial community assembly. Thus, out of the 353 most abundant bacterial OTUs across all treatments (i.e., relative abundance of at least 0.5% in any sample), the relative fitness of 139 OTUs was significantly stimulated in at least one of the removal treatments with 3- to more than 5000-fold changes compared to the NT control.

Because larger microorganisms were eliminated by filtering all soil suspensions at 10 μm before treatment and no known bacterivore bacterium was identified among the depleted OTUs, it is likely that exploitative or interference competition was responsible for the inferred negative interactions even though predation and parasitism cannot be ruled out [62]. Some of these bacterial OTUs were abundant in one or more removal treatments, whereas they were barely detected in the NT control (Fig. 2). For example, a quarter of the most dominant OTUs in the oxidative stress treatment belonged to the Bacillales, which accounted for 21% of the bacterial community, whereas Bacillales were rare in both the NT control and the original soil samples (0.57% and 0.14%, respectively). This was the case even when considering that the total bacterial abundance was two to six times lower in the oxidative stress treatment than in the original soil samples or in the NT control (Supplementary Fig. 8). This increase in the proportion of Bacillales in the community, usually a low-abundance taxa in soils [63], supports the idea that microbial rank abundance curves may be highly dynamic and that even rare subordinate species can become dominant when conditions turn out to be more favorable [64, 65]. Given that environmental factors and resources were the same for all microcosms before inoculation, our findings indicate that competitive exclusion rather than abiotic constraints prevents the rise of rare populations during soil recolonization. One interesting question is why high fitness inequalities have not driven the weak competitors to extinction in the original soil samples, a contradiction known as the biodiversity paradox. In the case of the Bacillales, it is likely their ability to enter a dormant state as spores and therefore to persist in a non-interactive state that explains their coexistence over time via the soil spore bank as previously suggested using theoretical models [64]. Overall, our findings in complex soil systems show that 39% of the dominant bacterial taxa across treatments were subjected to competitive interactions during soil recolonization. Although our approach may have inflated biotic interactions, for example by depriving bacteria from their natural shelters, the results extend previous evidence from in vitro and theoretical studies suggesting that competition is a common type of biotic interaction between bacteria [22, 62, 66]. Despite being beyond the scope of this work, efforts to characterize the mechanistic forces behind the observed interactions will allow deciphering whether exploitative or interference competition was prevalent in the studied soil microbiota.

### Ecological network inference recapitulates biotic interactions

**Fig. 4:**
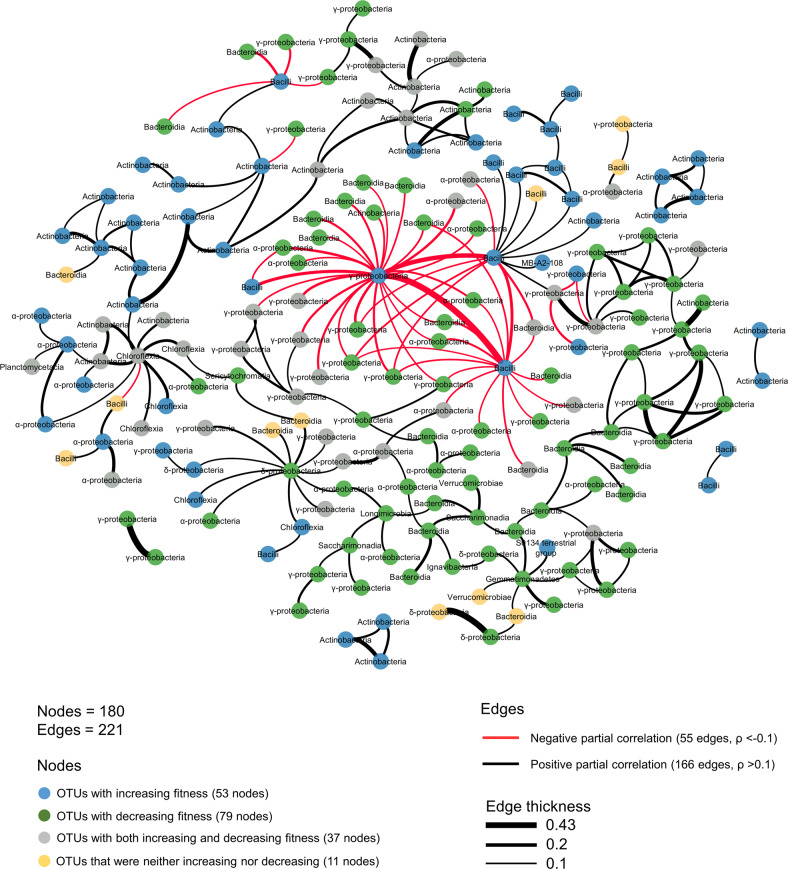
Bacterial network inferred using all treatments and NT control samples. The nodes represent individual OTUs with the corresponding phylum. The nodes are also colored according to the DESeq2 results: green nodes represent OTUs with decreasing fitness, blue nodes represent OTUs with increasing fitness, gray nodes represent OTUs with both increasing and decreasing fitness (depending on the treatment), and orange nodes represent OTUs that were neither increasing nor decreasing. Edge thickness is proportional to partial correlations between nodes and represents associative (black, *ρ* > 0.1) or exclusionary relationships (red, *ρ* < −0.1).

We reconstructed different networks, to infer significant associations between microorganisms, using a recently developed sparse multivariate PLN model [49]. First, the inferred global network across domains from all manipulation treatments and the NT control shows no association between bacteria and fungi, while significant edges between both domains were observed in the original soil samples (Supplementary Fig. 9a, b, respectively). This supports the results of the DESeq2 analysis, suggesting that bacteria rather than fungi were engaged in interactions unraveled by our removal approach. Next, we inferred a bacterial network including all treatments. To identify bacterial OTUs whose depletion actually leads to a significant increased relative fitness of the remaining ones, the nodes were colored according to the results of the DESeq2 analysis (Fig. 4). Out of the 180 OTUs present in this network, 90 were among those that significantly increased in at least one of the removal treatments compared to the NT control. Moreover, 50 out of 55 negative edges in the network are connecting depleted OTUs to OTUs with increased relative fitness. Thus, the interactions unraveled by our microbial community manipulation approach closely matched the inferred nodes and edges, which provides a cross-validation of our network analysis. Using a removal approach allowed going beyond simply testing significant associations in natural communities, as we could determine which OTUs were outcompeted. Thus, we found conserved negative interactions indicating that Bacillales were capable to grow only when α− and/or γ- Proteobacteria were depleted by the removal treatments (e.g., heat shock and oxidative stress; Figs. 2 and 4). Similar antagonistic patterns have been observed in previous studies showing segregated spatial co-occurrences or checkerboard patterns between Firmicutes and γ- Proteobacteria [55, 67]. Interestingly, in the global bacterial network, several γ-Proteobacteria (Xanthomonas sp.) have negative edges with another γ-Proteobacteria (Burkholderia sp.) and two Bacillus hubs, which were themselves connected by negative edges. Since the fitness of these Bacillus and Burkholderia strains was significantly increased by a removal treatment, this suggests that these strains were thriving, but in competition when their competitors were depleted. Altogether these findings can be interpreted as evidence for predictable rule of bacterial community assembly. Such understanding of what govern community assembly would be instrumental for steering the soil microbiome toward a community that enhance ecosystem services [9, 20].

### Shifts in soil functions associated with microbial community manipulation

Soil functions related to the C and N cycles were investigated using microbial respiration rates of different C substrates (MicroResp), inorganic nitrogen pools as well as the abundance of ammonia-oxidizer and denitrifier genes as proxies for these specific processes. We found significant differences in the emergent functions of the manipulated soil microbial communities (Tukey’s test, *p* value < 0.05; Supplementary Fig. 10). Notably, microbial communities that were subjected to ciprofloxacin exhibited a significantly higher ability to respire glucosamine and fructose than all other removal treatments and controls (Tukey’s test, *p* value < 0.05; Supplementary Fig. 11), whereas respiration rates of many C substrates were lower in microbial communities that assembled after heat shock and oxidative stress treatments. Among the soil functions assessed, the greatest changes were observed for N cycling. The soil nitrate content declined in all treatments except one (0.8 µm filtration) when compared to the original soil samples (Tukey’s test, *p* value < 0.05; Supplementary Fig. 10b), which is in accordance with a previous manipulation experiment [55].

**Fig. 5:**
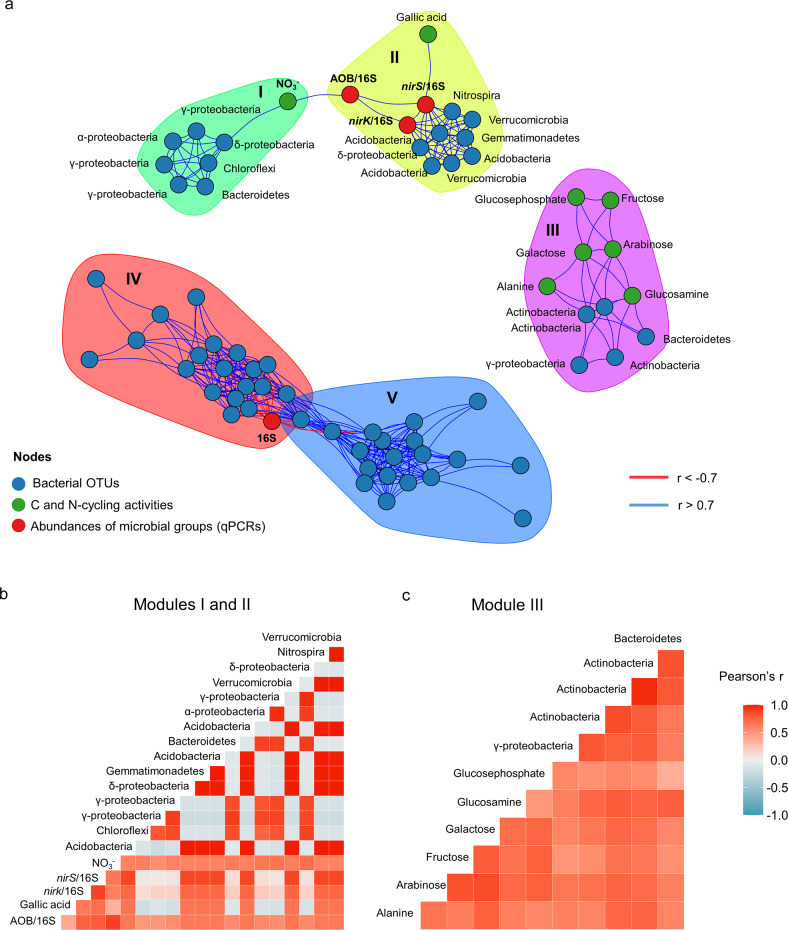
Data integration analysis for the identification of highly correlated variables across data sets. **a** Visualization of associated bacterial OTUs (blue nodes), N- and C-cycling activities (green nodes), and abundances of microbial groups (red nodes). The taxonomic identities of the OTUs are indicated at the phylum or class level. The N- and C-cycling activities are based on inorganic N pools and respiration rates of different substrates, respectively. Abundances of microbial groups are corresponding to the total bacterial community abundance (16S), the proportion of ammonia-oxidizing bacteria, and denitrifiers in the total bacterial community (AOB/16S, *nirK*/16S, and *nirS*/16S, respectively). Edges indicate positive (blue) or negative correlations (red) as defined by Pearson’s correlation *r* > 0.7 or *r* < −0.7, respectively. Different modules are represented by different colors and numbered (I–V). Correlation matrices of variables across data types in **b** modules I and II (green and yellow) and **c** within module III (purple).

To further explore the relationship between the composition of the manipulated microbial community membership and soil functions, we used a multivariate dimension reduction discriminant analysis method that builds on Projection to Latent Structure models [54] (Fig. 5). The red and blue modules are showing OTUs (mostly Firmicutes and Actinobacteria and Actinobacteria, Chloroflexi and α-Proteobacteria, respectively) that were highly correlated with each other and with the total bacterial abundance but without any correlation to a soil function. However, several correlations that emerged in the other modules agreed with empirical knowledge. For example, Actinobacteria, which have a well-described role in carbon cycling [68], are representing three out of the five bacterial OTUs that were grouped in the module containing all the C-substrate respiration rates but gallic acid. In line with previous studies describing soil nitrification—the oxidation of ammonia to nitrate—as usually limited by its first step performed by ammonia oxidizers [69], the nitrate pool was correlated with the proportion of ammonia-oxidizing bacteria in the total bacterial community (AOB/16S rRNA). The proportion of AOB was included in a fully connected module comprising the proportion of *nirK*- and *nirS*-denitrifiers and eight OTUs, one of them belonging to the *Nitrospiracea*, a well-known group of nitrite oxidizers [70]. Interestingly, the nitrate pool was also correlated with another module of seven OTUs, one of them belonging to the Chloroflexi, which have been identified as a novel group of nitrite oxidizing bacteria [71] (Fig. 5). Since the nitrate pool and the abundance of AOB were the highest both in the original soil samples and in the 0.8 µm filtration treatment, while 16S rRNA gene sequences from known bacterial nitrifiers (*Nitrospira* and *Nitrosomonas*) in the 0.8 µm filtration treatment was about eight times lower than in original soil samples, one can speculate that one or several of these OTUs in this module could be also be capable of ammonia oxidation.

## Conclusions

By exploring biotic interactions within a community of naturally co-occurring soil microorganisms during the recolonization of their original habitat, we demonstrated that 39% of the dominant bacteria across treatments were subjected to negative interactions during community assembly. The approach used here allowed us to tie correlation patterns inferred by network analysis to ecological interactions revealed by experimental manipulation of the microbial community. We found evidence for competitive interactions between members of the low-abundance Bacillales and the dominant Proteobacteriales and suggest that competition-driven niche segregation rather than habitat features prevents the rise of rare populations in soil. Differences in the emergent functions of the manipulated communities were detected, with more pronounced shifts in functions related to N- rather than C-cycling. Thus, microbial community manipulation by removal, in addition to being informative about biotic interactions during assembly, may represent an alternative avenue to better understand the links between microbial community composition and ecosystem functioning based on the analogy to gene-knockout procedures in genomics [72]. Overall, our results suggest that some simple rules of bacterial community assembly can be identified, which has potential for predicting and steering the soil microbiota to promote or suppress certain functions in managed ecosystems. However, whether these empirically observed interactions can be generalized to other environments remains to be elucidated.

## Supplementary information

Supplementary Material
