# Supplementary material for: Unraveling negative biotic interactions determining soil microbial community assembly and functioning
Source: ISME J. 2021 Jul 28;16(1):296–306. doi: 10.1038/s41396-021-01076-9 (PMC8692615; doi:10.1038/s41396-021-01076-9)
Supplement: Supplementary file 1 — Supplementary Material [file 41396_2021_1076_MOESM1_ESM.pptx]

## Slide 1
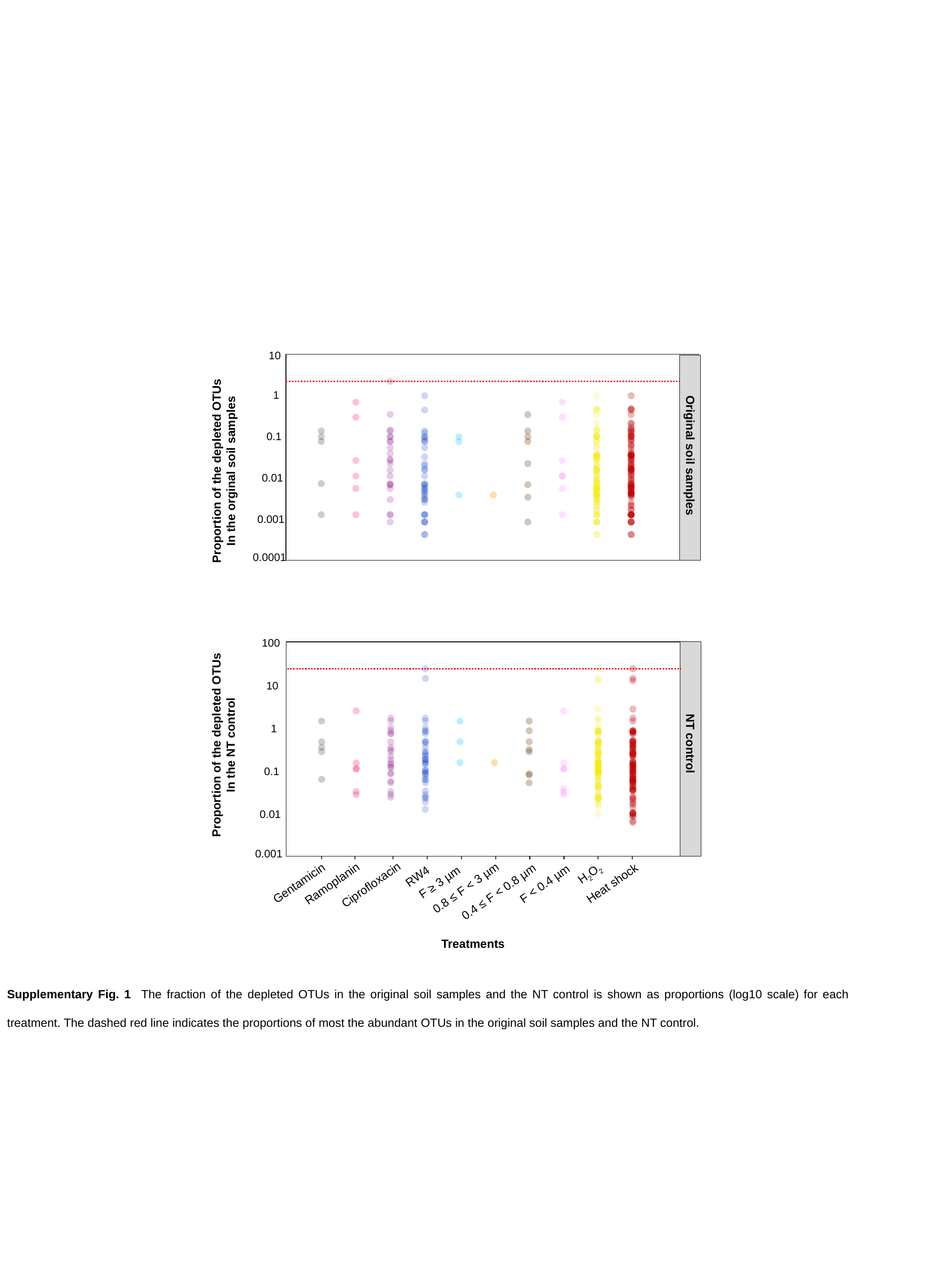

10
1
0.1
Original soil samples
0.01
0.001
0.0001
Proportion of the depleted OTUs
In the orginal soil samples
100
10
1
Proportion of the depleted OTUs
In the NT control
NT control
0.1
0.01
0.001
H2O2
RW4
F ≥ 3 µm
Ramoplanin
Gentamicin
Heat shock
F < 0.4 µm
Ciprofloxacin
0.4 ≤ F < 0.8 µm
0.8 ≤ F < 3 µm
Treatments
Supplementary Fig. 1 The fraction of the depleted OTUs in the original soil samples and the NT control is shown as proportions (log10 scale) for each treatment. The dashed red line indicates the proportions of most the abundant OTUs in the original soil samples and the NT control.

## Slide 2
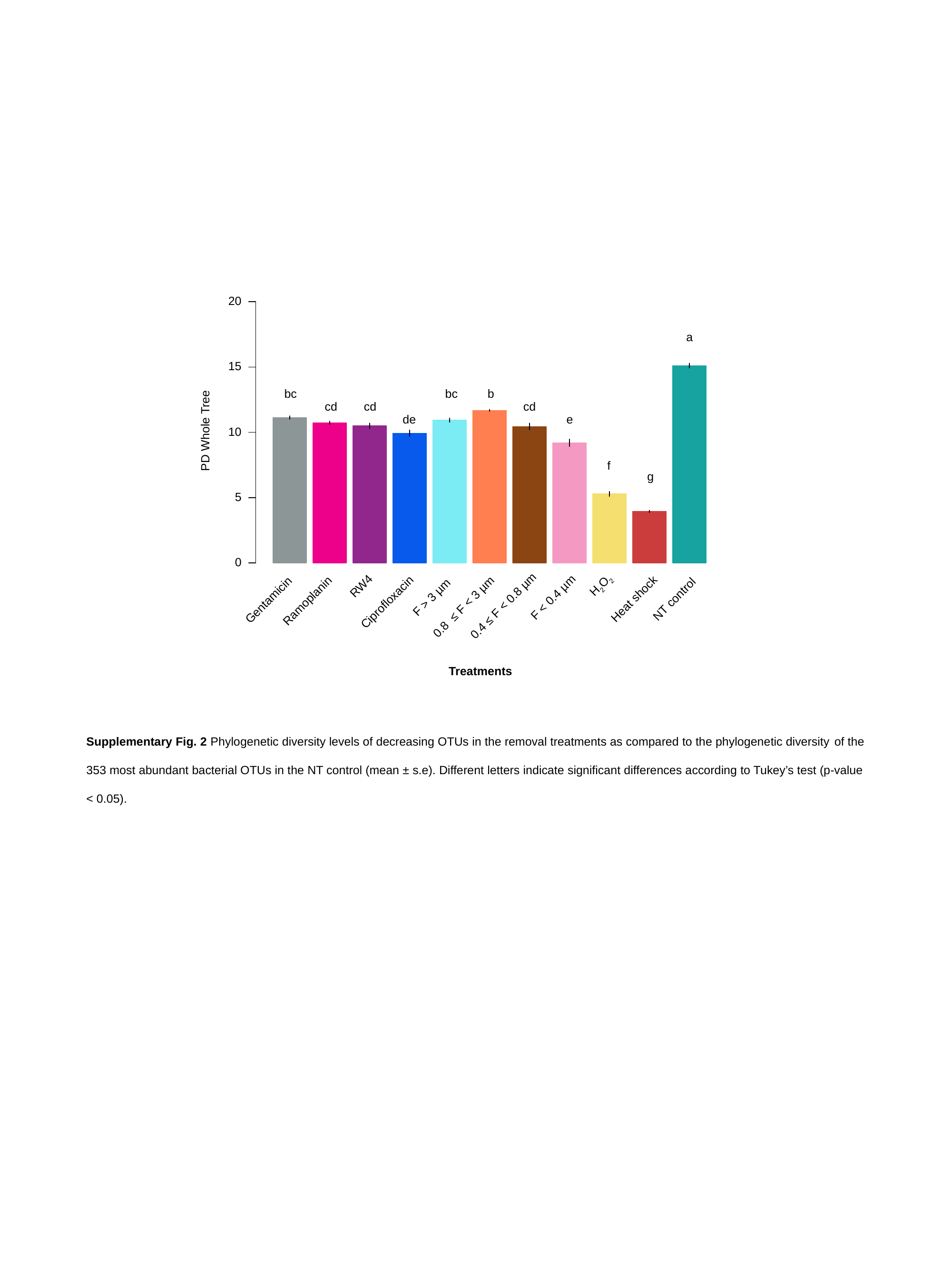

20
15
10
 PD Whole Tree
5
0
RW4
H2O2
F > 3 µm
F < 0.4 µm
Heat shock
NT control
Ramoplanin
Gentamicin
Ciprofloxacin
0.4 ≤ F < 0.8 µm
0.8 ≤ F < 3 µm
Treatments
a
bc
bc
b
cd
cd
cd
de
e
f
g
Supplementary Fig. 2 Phylogenetic diversity levels of decreasing OTUs in the removal treatments as compared to the phylogenetic diversity of the 353 most abundant bacterial OTUs in the NT control (mean ± s.e). Different letters indicate significant differences according to Tukey’s test (p-value < 0.05).

## Slide 3
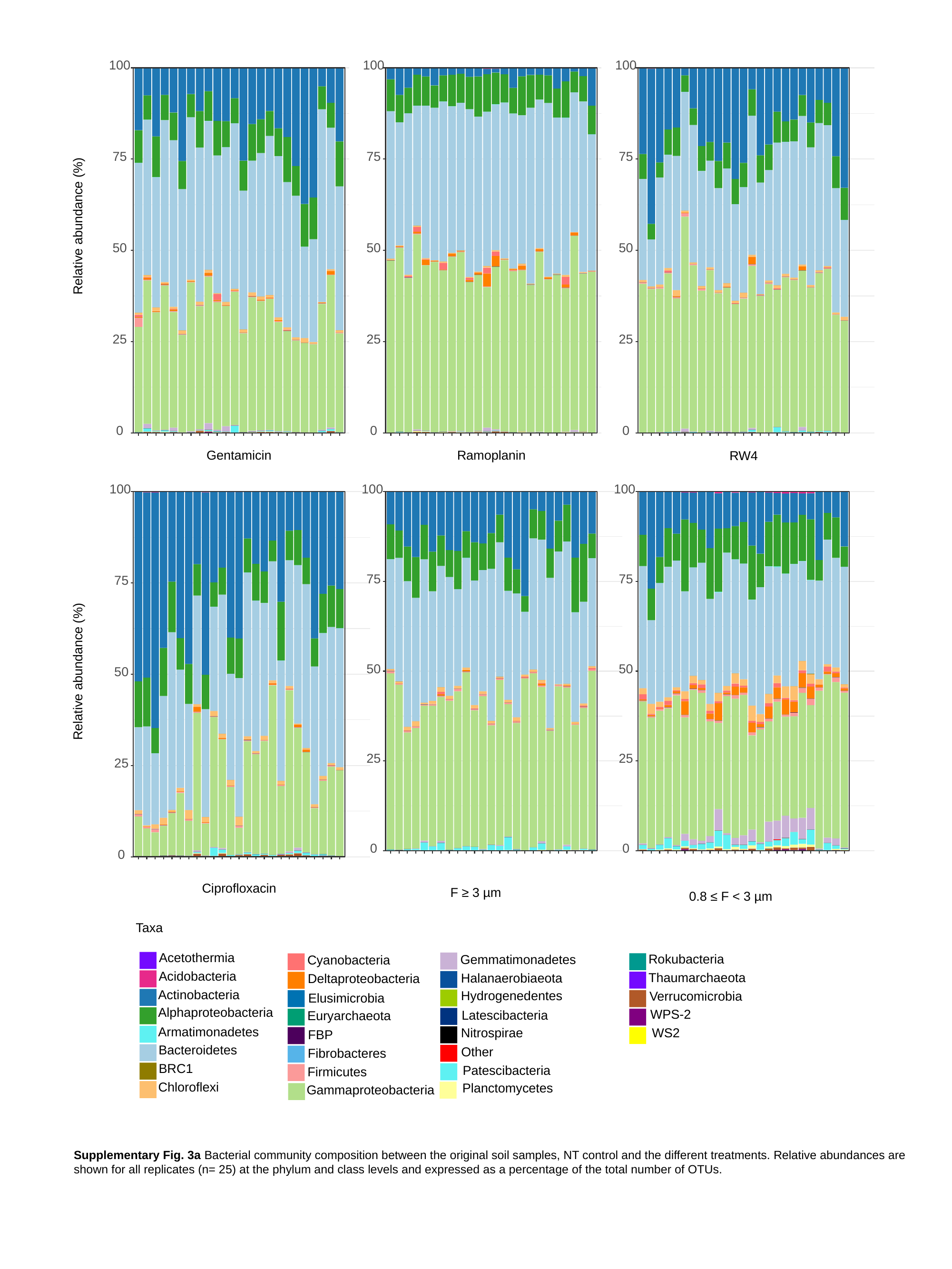

100
100
100
75
75
75
Relative abundance (%)
50
50
50
25
25
25
0
0
0
Gentamicin
Ramoplanin
RW4
100
100
100
75
75
75
50
50
Relative abundance (%)
50
25
25
25
0
0
0
Ciprofloxacin
0.8 ≤ F < 3 µm
F ≥ 3 µm
Taxa
Acetothermia
Rokubacteria
Cyanobacteria
Gemmatimonadetes
Acidobacteria
Thaumarchaeota
Deltaproteobacteria
Halanaerobiaeota
Actinobacteria
Hydrogenedentes
Verrucomicrobia
Elusimicrobia
Alphaproteobacteria
WPS-2
Euryarchaeota
Latescibacteria
Armatimonadetes
Nitrospirae
WS2
FBP
Bacteroidetes
Other
Fibrobacteres
BRC1
Patescibacteria
Firmicutes
Chloroflexi
Planctomycetes
Gammaproteobacteria
Supplementary Fig. 3a Bacterial community composition between the original soil samples, NT control and the different treatments. Relative abundances are shown for all replicates (n= 25) at the phylum and class levels and expressed as a percentage of the total number of OTUs.

## Slide 4
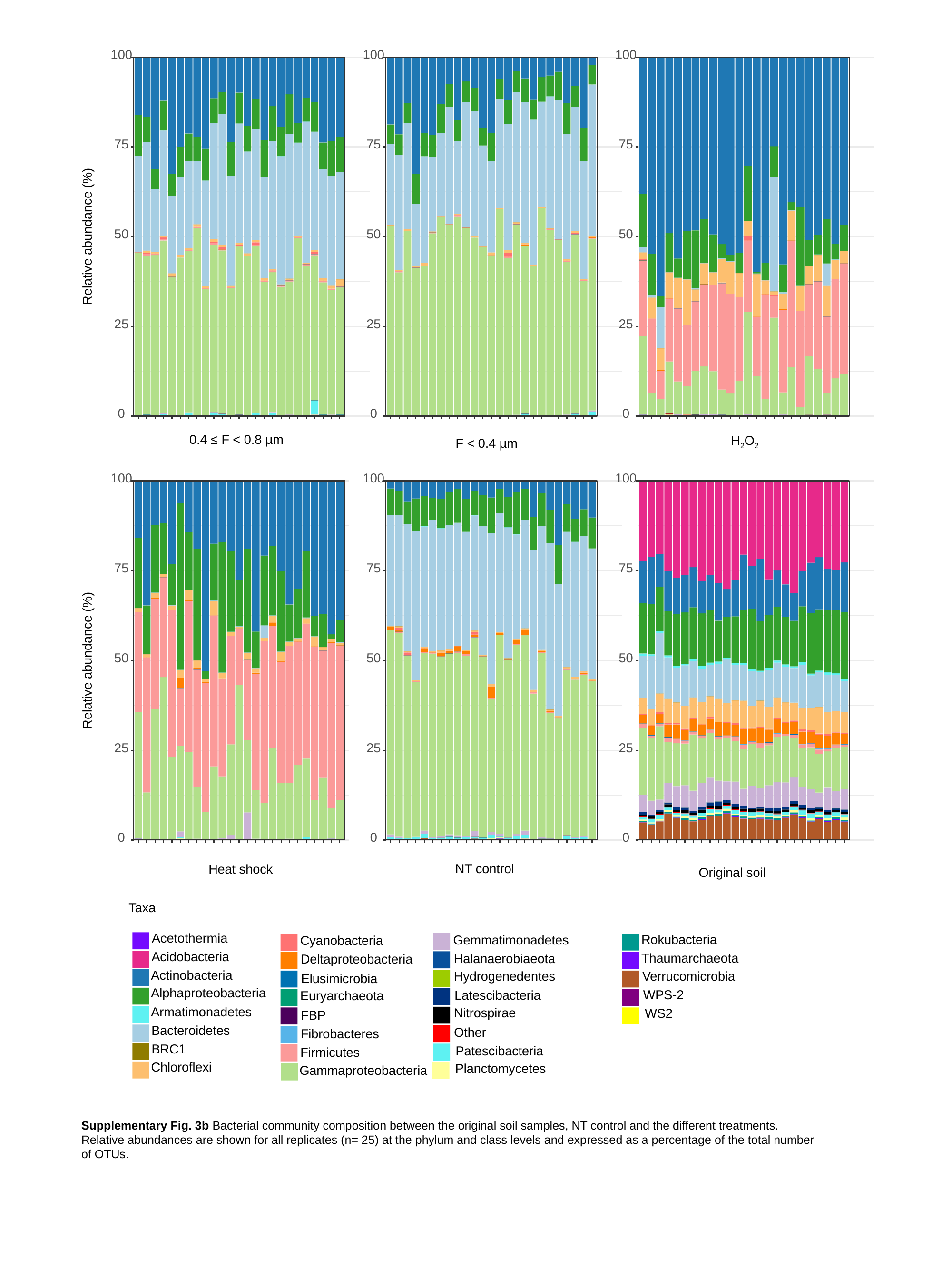

100
100
100
75
75
75
50
50
50
Relative abundance (%)
25
25
25
H2O2
0
0
0
0.4 ≤ F < 0.8 µm
F < 0.4 µm
100
100
100
75
75
75
50
50
50
Relative abundance (%)
25
25
25
0
0
0
Heat shock
Original soil
NT control
Taxa
Acetothermia
Rokubacteria
Cyanobacteria
Gemmatimonadetes
Acidobacteria
Thaumarchaeota
Deltaproteobacteria
Halanaerobiaeota
Actinobacteria
Hydrogenedentes
Verrucomicrobia
Elusimicrobia
Alphaproteobacteria
WPS-2
Euryarchaeota
Latescibacteria
Armatimonadetes
Nitrospirae
WS2
FBP
Bacteroidetes
Other
Fibrobacteres
BRC1
Patescibacteria
Firmicutes
Chloroflexi
Planctomycetes
Gammaproteobacteria
Supplementary Fig. 3b Bacterial community composition between the original soil samples, NT control and the different treatments. Relative abundances are shown for all replicates (n= 25) at the phylum and class levels and expressed as a percentage of the total number of OTUs.

## Slide 5
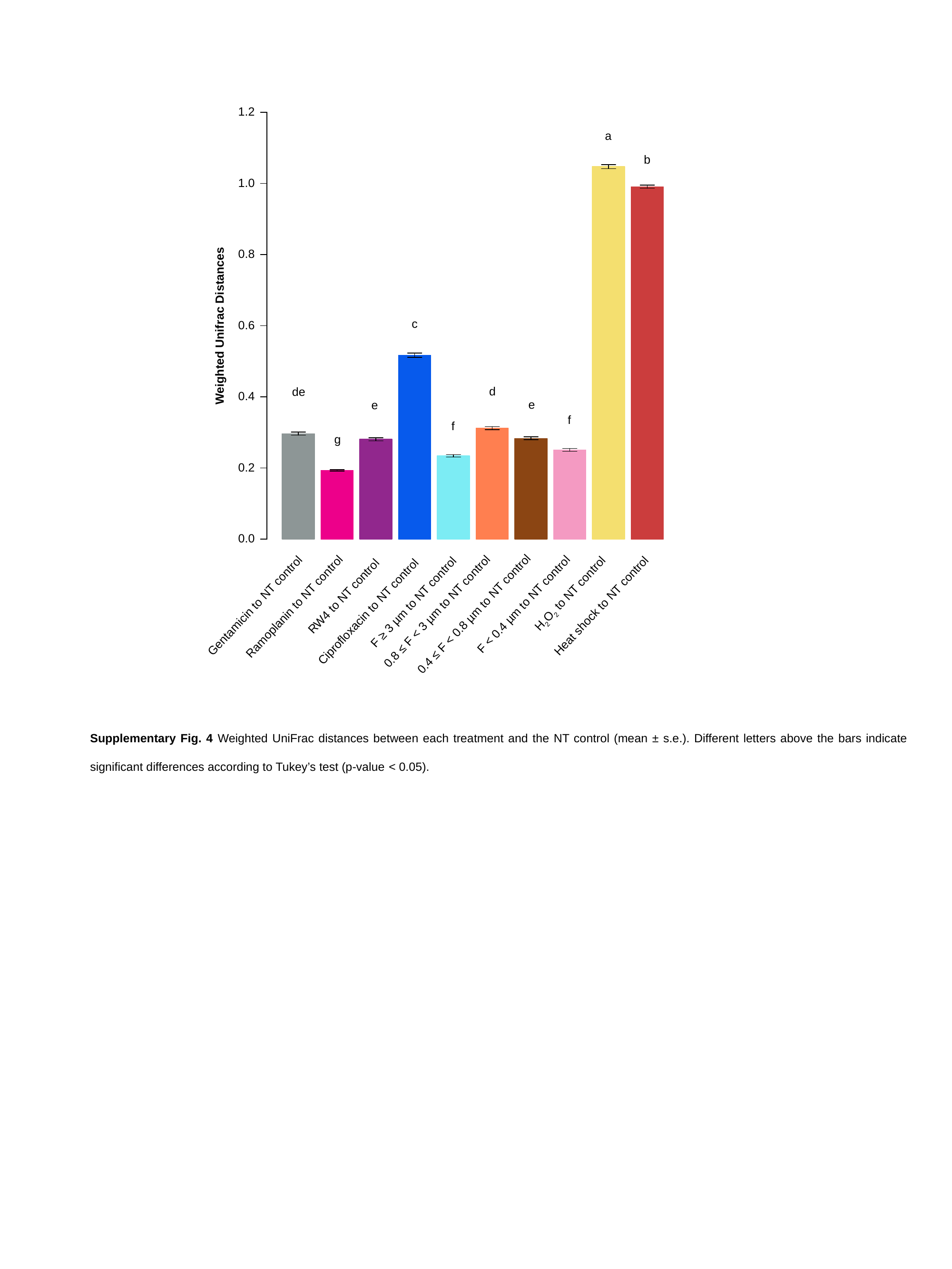

1.2
a
b
1.0
0.8
c
Weighted Unifrac Distances
0.6
d
de
0.4
e
e
f
f
g
0.2
0.0
H2O2 to NT control
RW4 to NT control
F ≥ 3 µm to NT control
F < 0.4 µm to NT control
Gentamicin to NT control
Heat shock to NT control
Ramoplanin to NT control
0.8 ≤ F < 3 µm to NT control
Ciprofloxacin to NT control
0.4 ≤ F < 0.8 µm to NT control
Supplementary Fig. 4 Weighted UniFrac distances between each treatment and the NT control (mean ± s.e.). Different letters above the bars indicate significant differences according to Tukey’s test (p-value < 0.05).

## Slide 6
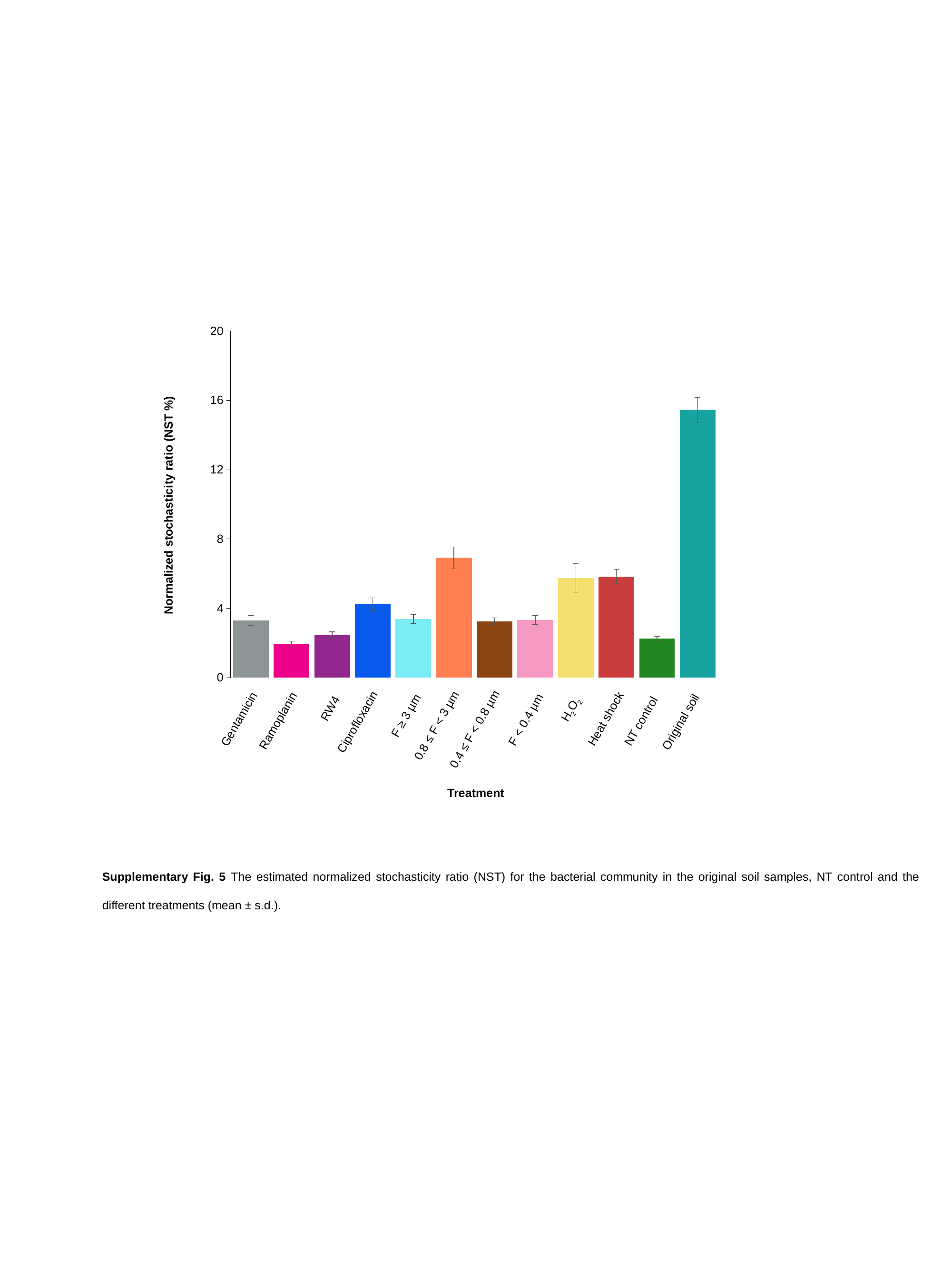

### Chart
| Category | obs.NST |
|---|---|
| Gentamicin | 3.30067925173798 |
| Ramoplanin | 1.9535413260974799 |
| RW4 | 2.4594516365167003 |
| Ciprofloxacin | 4.22557065399809 |
| Filtre3um | 3.38457148068484 |
| Filtre0_8um | 6.9154675826200895 |
| Filtre0_4um | 3.2508597501700396 |
| Filtrat0_4um | 3.3324974123449502 |
| H2O2 | 5.75031601521777 |
| Heat | 5.83164974960743 |
| ControlT45 | 2.26792074766017 |
| ControlT0 | 15.448020916518301 |Normalized stochasticity ratio (NST %)
RW4
H2O2
F ≥ 3 µm
Gentamicin
Ramoplanin
F < 0.4 µm
0.4 ≤ F < 0.8 µm
Heat shock
Original soil
Ciprofloxacin
NT control
0.8 ≤ F < 3 µm
Treatment
Supplementary Fig. 5 The estimated normalized stochasticity ratio (NST) for the bacterial community in the original soil samples, NT control and the different treatments (mean ± s.d.).

## Slide 7
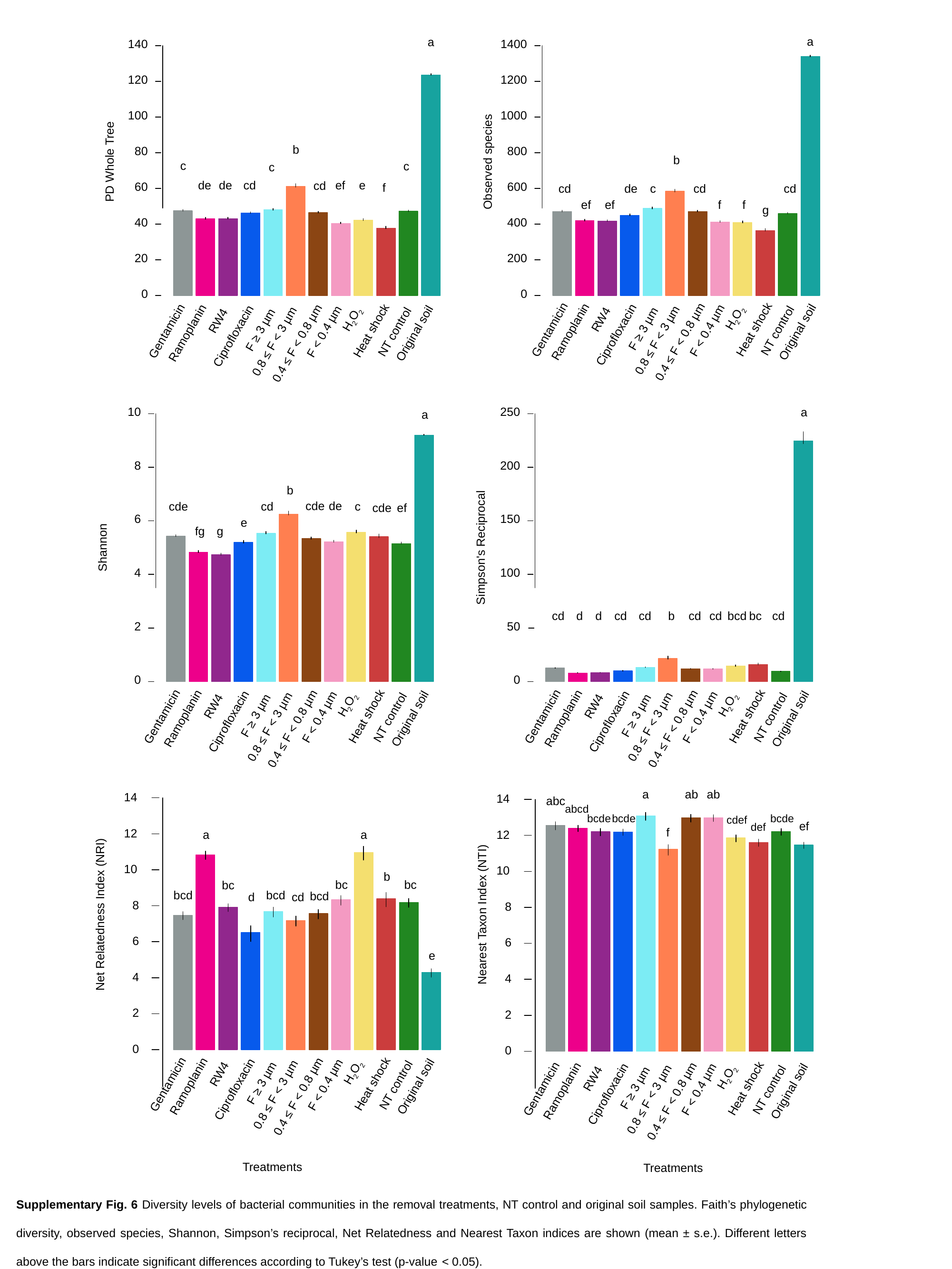

a
a
140
1400
120
1200
100
1000
b
80
800
b
c
c
Observed species
c
PD Whole Tree
e
ef
cd
de
de
cd
f
cd
de
c
cd
cd
60
600
ef
ef
f
f
g
40
400
20
200
H2O2
RW4
F ≥ 3 µm
F < 0.4 µm
Heat shock
Gentamicin
Ramoplanin
NT control
Original soil
Ciprofloxacin
0.8 ≤ F < 3 µm
0.4 ≤ F < 0.8 µm
H2O2
RW4
F ≥ 3 µm
F < 0.4 µm
Heat shock
Gentamicin
Ramoplanin
NT control
Original soil
Ciprofloxacin
0.8 ≤ F < 3 µm
0.4 ≤ F < 0.8 µm
0
0
a
a
10
250
8
200
b
cde
de
cde
cd
c
cde
ef
e
6
150
fg
g
Simpson's Reciprocal
Shannon
100
4
cd
d
d
cd
cd
b
cd
cd
bcd
bc
cd
50
2
H2O2
RW4
F ≥ 3 µm
F < 0.4 µm
Heat shock
Gentamicin
Ramoplanin
NT control
Original soil
Ciprofloxacin
0.8 ≤ F < 3 µm
0.4 ≤ F < 0.8 µm
H2O2
RW4
F ≥ 3 µm
F < 0.4 µm
Heat shock
Gentamicin
Ramoplanin
NT control
Original soil
Ciprofloxacin
0.8 ≤ F < 3 µm
0.4 ≤ F < 0.8 µm
0
0
a
ab
ab
abc
abcd
bcde
bcde
bcde
cdef
ef
def
f
H2O2
RW4
F ≥ 3 µm
F < 0.4 µm
Heat shock
Gentamicin
Ramoplanin
NT control
Original soil
Ciprofloxacin
0.8 ≤ F < 3 µm
0.4 ≤ F < 0.8 µm
14
14
a
a
12
12
b
10
10
bc
bc
bc
bcd
bcd
bcd
d
cd
8
8
Net Relatedness Index (NRI)
 Nearest Taxon Index (NTI)
6
6
e
4
4
2
2
H2O2
RW4
F ≥ 3 µm
F < 0.4 µm
Heat shock
Gentamicin
Ramoplanin
NT control
Original soil
Ciprofloxacin
0.8 ≤ F < 3 µm
0.4 ≤ F < 0.8 µm
0
0
Treatments
Treatments
Supplementary Fig. 6 Diversity levels of bacterial communities in the removal treatments, NT control and original soil samples. Faith’s phylogenetic diversity, observed species, Shannon, Simpson’s reciprocal, Net Relatedness and Nearest Taxon indices are shown (mean ± s.e.). Different letters above the bars indicate significant differences according to Tukey’s test (p-value < 0.05).

## Slide 8
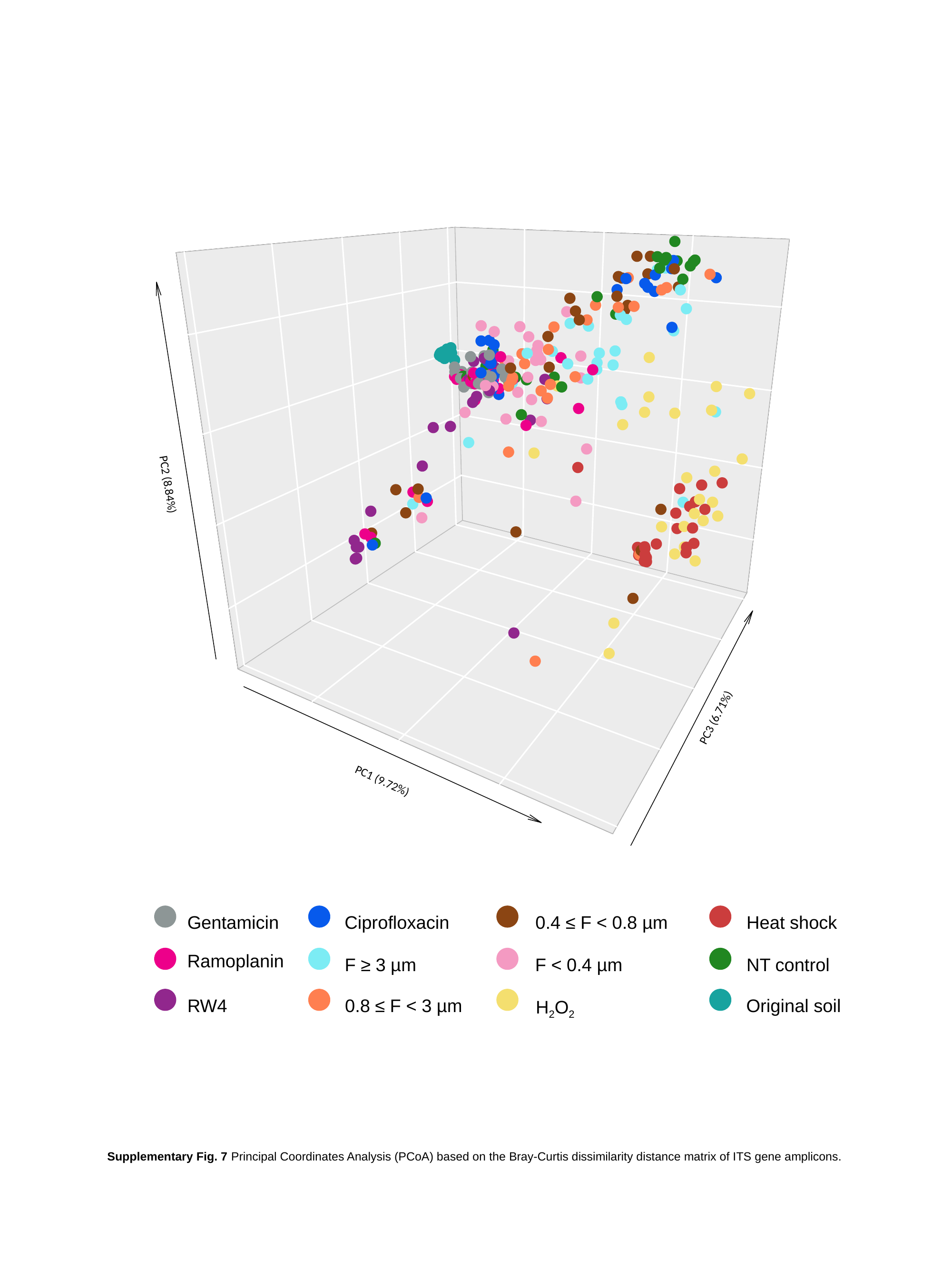

PC2 (8.84%)
PC3 (6.71%)
PC1 (9.72%)
Gentamicin
Ciprofloxacin
0.4 ≤ F < 0.8 µm
Heat shock
Ramoplanin
F ≥ 3 µm
F < 0.4 µm
NT control
RW4
0.8 ≤ F < 3 µm
H2O2
Original soil
Supplementary Fig. 7 Principal Coordinates Analysis (PCoA) based on the Bray-Curtis dissimilarity distance matrix of ITS gene amplicons.

## Slide 9
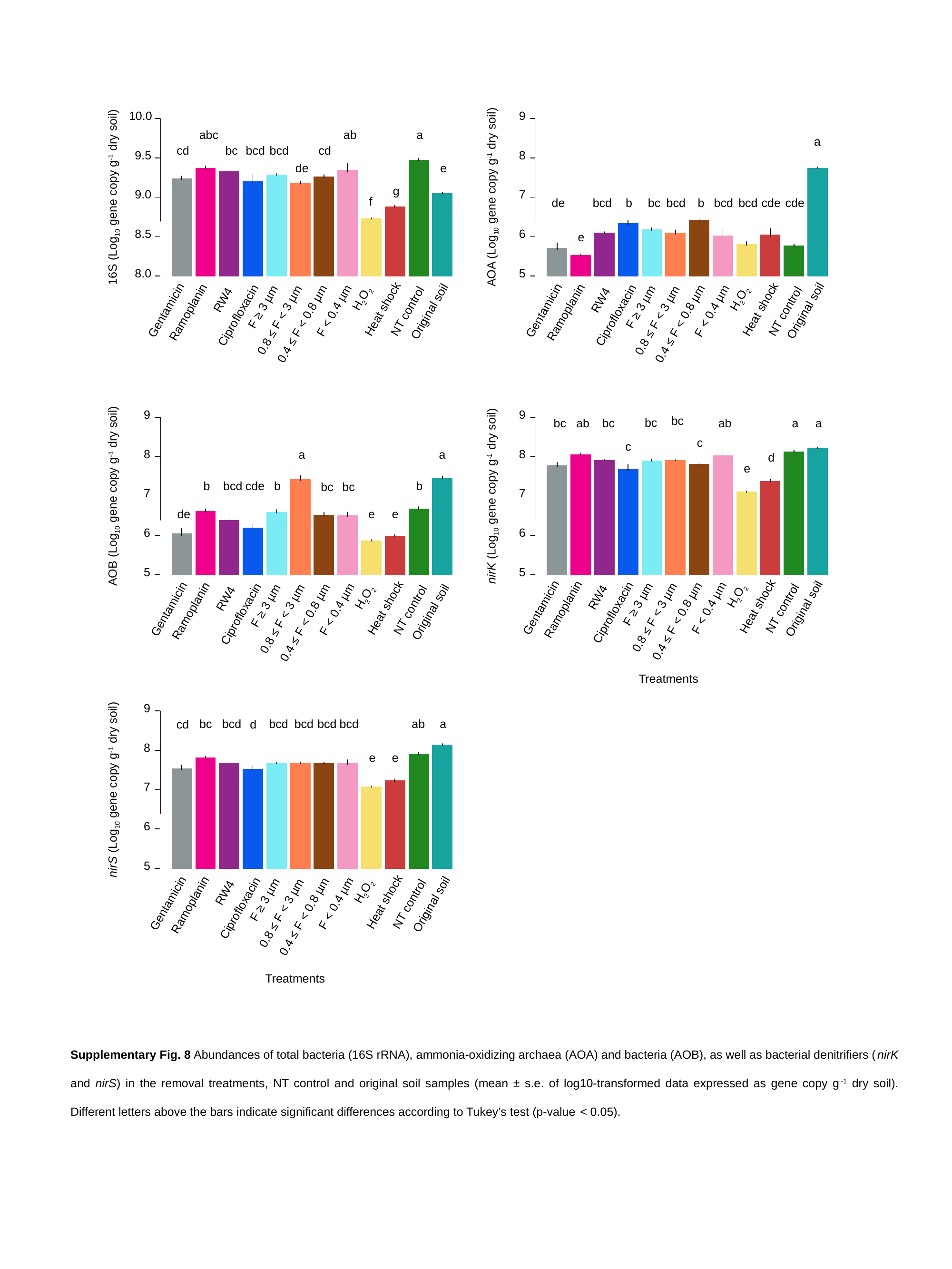

10.0
9
abc
ab
a
a
cd
bc
bcd
bcd
cd
9.5
8
de
e
g
f
de
bcd
b
bc
bcd
b
bcd
bcd
cde
cde
16S (Log10 gene copy g-1 dry soil)
AOA (Log10 gene copy g-1 dry soil)
9.0
7
e
8.5
6
H2O2
RW4
F ≥ 3 µm
Heat shock
Gentamicin
F < 0.4 µm
Ramoplanin
NT control
Original soil
Ciprofloxacin
0.8 ≤ F < 3 µm
0.4 ≤ F < 0.8 µm
H2O2
RW4
F ≥ 3 µm
Heat shock
Gentamicin
Original soil
F < 0.4 µm
Ramoplanin
NT control
Ciprofloxacin
0.8 ≤ F < 3 µm
0.4 ≤ F < 0.8 µm
8.0
5
bc
bc
bc
ab
bc
ab
a
a
9
9
c
c
a
a
d
8
8
e
bcd
cde
b
b
b
bc
bc
AOB (Log10 gene copy g-1 dry soil)
nirK (Log10 gene copy g-1 dry soil)
7
7
de
e
e
6
6
H2O2
RW4
F ≥ 3 µm
Heat shock
Gentamicin
F < 0.4 µm
Ramoplanin
NT control
Ciprofloxacin
0.8 ≤ F < 3 µm
0.4 ≤ F < 0.8 µm
H2O2
RW4
F ≥ 3 µm
Heat shock
Gentamicin
F < 0.4 µm
Ramoplanin
NT control
Original soil
Ciprofloxacin
0.8 ≤ F < 3 µm
0.4 ≤ F < 0.8 µm
5
5
Original soil
Treatments
9
bc
bcd
bcd
bcd
bcd
bcd
ab
a
cd
d
8
e
e
nirS (Log10 gene copy g-1 dry soil)
7
6
H2O2
RW4
F ≥ 3 µm
Heat shock
Gentamicin
F < 0.4 µm
Ramoplanin
NT control
Original soil
Ciprofloxacin
0.8 ≤ F < 3 µm
0.4 ≤ F < 0.8 µm
5
Treatments
Supplementary Fig. 8 Abundances of total bacteria (16S rRNA), ammonia-oxidizing archaea (AOA) and bacteria (AOB), as well as bacterial denitrifiers (nirK and nirS) in the removal treatments, NT control and original soil samples (mean ± s.e. of log10-transformed data expressed as gene copy g-1 dry soil). Different letters above the bars indicate significant differences according to Tukey’s test (p-value < 0.05).

## Slide 10
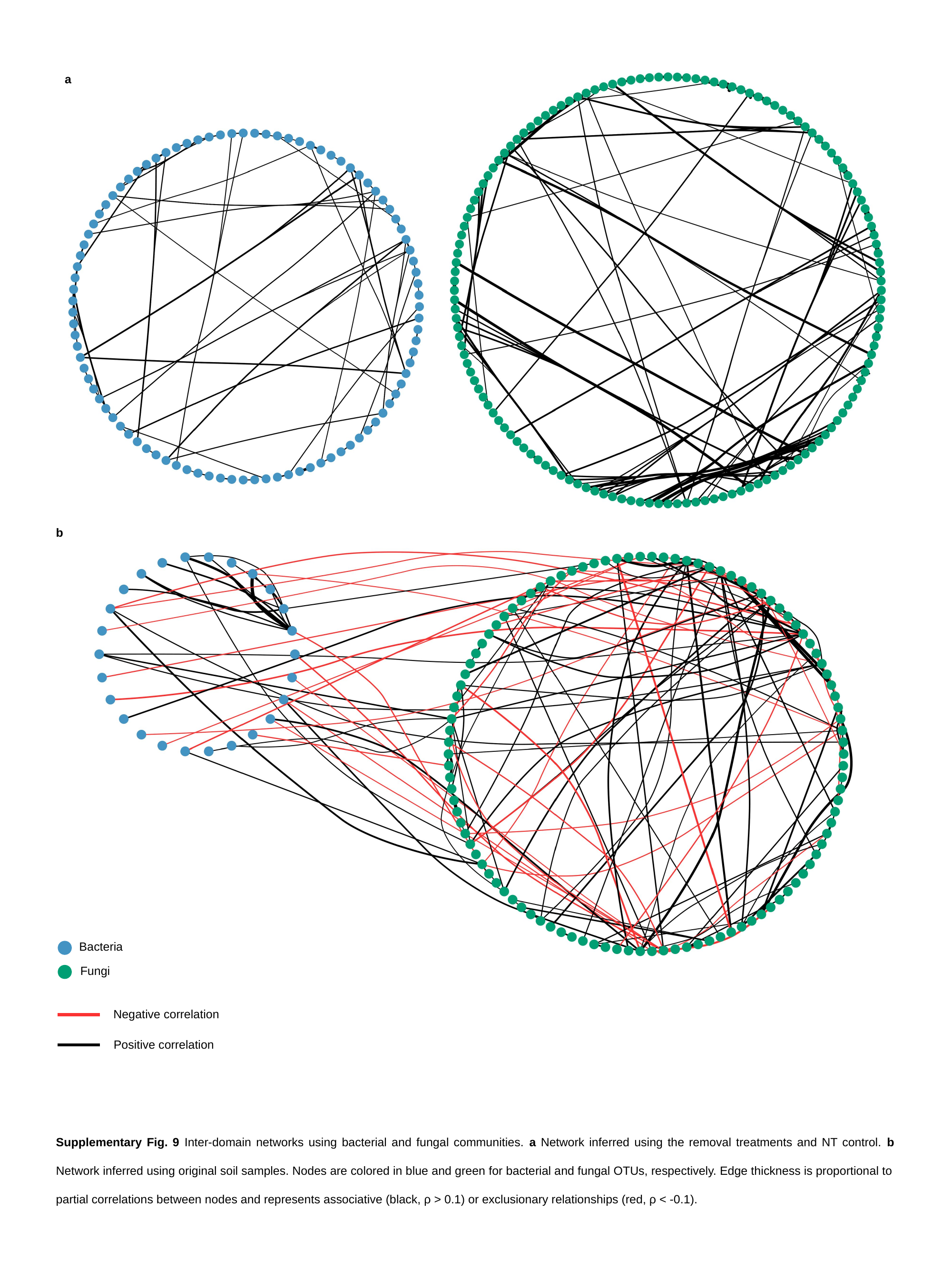

a
A
b
Bacteria
Fungi
Negative correlation
Positive correlation
Supplementary Fig. 9 Inter-domain networks using bacterial and fungal communities. a Network inferred using the removal treatments and NT control. b Network inferred using original soil samples. Nodes are colored in blue and green for bacterial and fungal OTUs, respectively. Edge thickness is proportional to partial correlations between nodes and represents associative (black, ρ > 0.1) or exclusionary relationships (red, ρ < -0.1).

## Slide 11
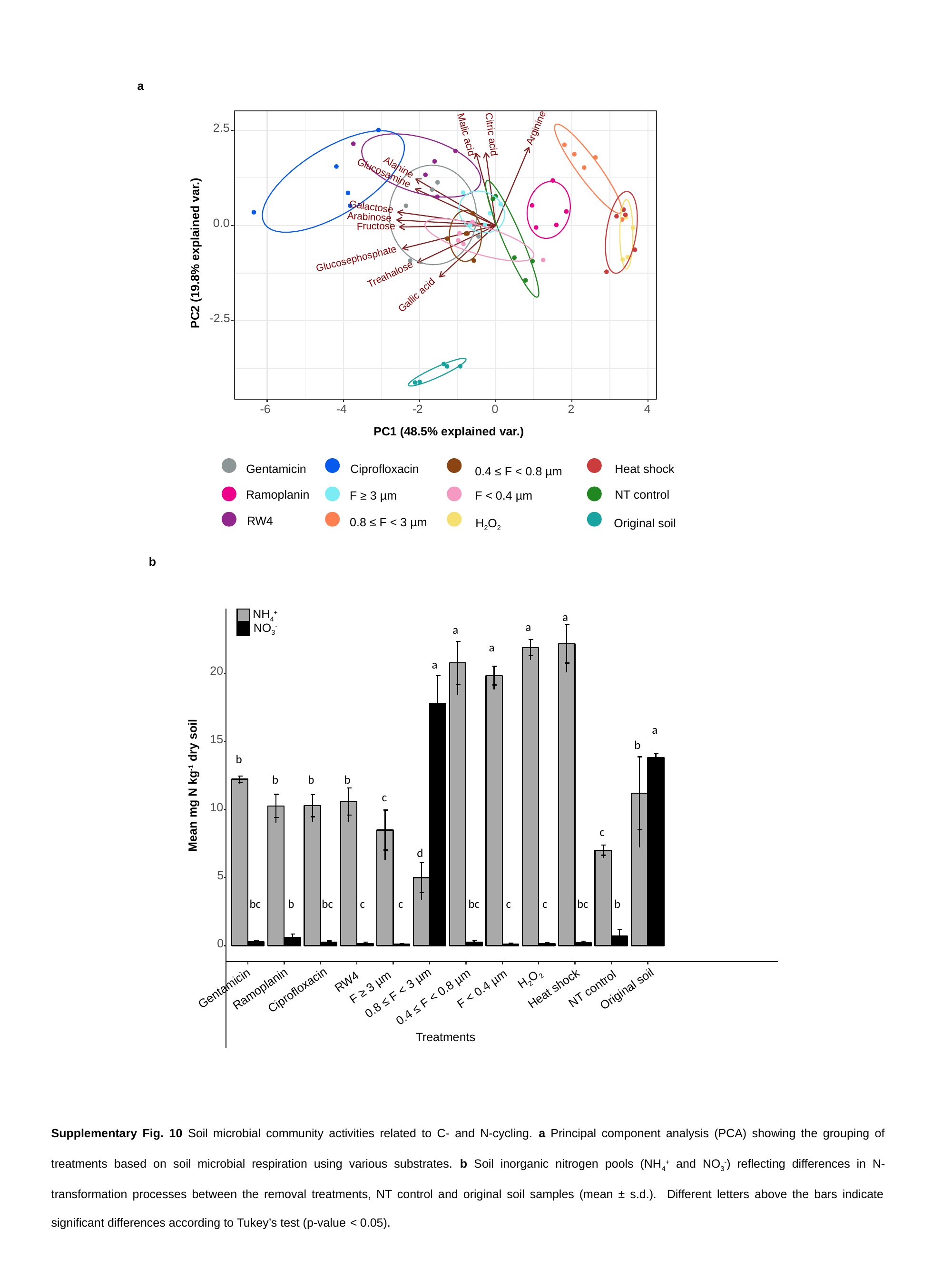

a
Arginine
2.5
Citric acid
Malic acid
Alanine
Glucosamine
Galactose
Arabinose
0.0
Fructose
PC2 (19.8% explained var.)
Glucosephosphate
Treahalose
Gallic acid
-2.5
-6
0
-2
2
-4
4
PC1 (48.5% explained var.)
Gentamicin
Ciprofloxacin
Heat shock
0.4 ≤ F < 0.8 µm
Ramoplanin
NT control
F ≥ 3 µm
F < 0.4 µm
RW4
0.8 ≤ F < 3 µm
H2O2
Original soil
b
a
a
NH4+
a
NO3-
a
a
20
a
b
15
b
b
b
b
Mean mg N kg-1 dry soil
c
10
c
d
5
bc
b
bc
c
c
bc
c
c
bc
b
0
H2O2
RW4
F ≥ 3 µm
Ramoplanin
Gentamicin
Heat shock
NT control
Original soil
F < 0.4 µm
Ciprofloxacin
0.4 ≤ F < 0.8 µm
0.8 ≤ F < 3 µm
Treatments
Supplementary Fig. 10 Soil microbial community activities related to C- and N-cycling. a Principal component analysis (PCA) showing the grouping of treatments based on soil microbial respiration using various substrates. b Soil inorganic nitrogen pools (NH4+ and NO3-) reflecting differences in N-transformation processes between the removal treatments, NT control and original soil samples (mean ± s.d.). Different letters above the bars indicate significant differences according to Tukey’s test (p-value < 0.05).

## Slide 12
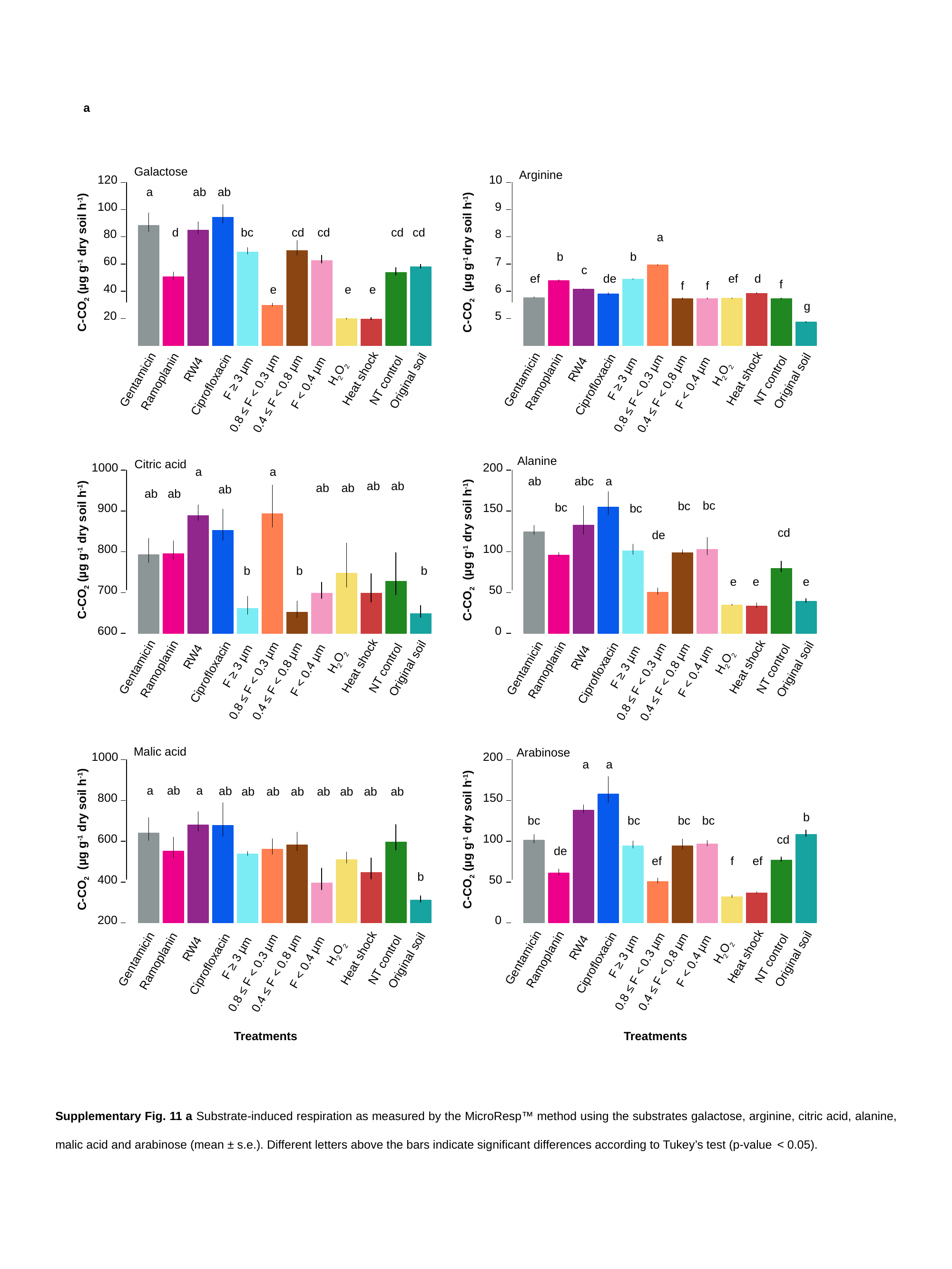

a
Galactose
Arginine
120
10
a
ab
ab
100
9
d
bc
cd
cd
cd
cd
a
80
8
b
b
c
 C-CO2 (µg g-1 dry soil h-1)
 C-CO2 (µg g-1 dry soil h-1)
60
7
ef
de
ef
d
f
f
f
e
e
e
40
6
g
20
5
RW4
H2O2
F ≥ 3 µm
Heat shock
Gentamicin
Ramoplanin
NT control
Original soil
Ciprofloxacin
0.8 ≤ F < 0.3 µm
0.4 ≤ F < 0.8 µm
F < 0.4 µm
RW4
H2O2
F ≥ 3 µm
Heat shock
Gentamicin
Ramoplanin
NT control
Original soil
Ciprofloxacin
0.8 ≤ F < 0.3 µm
0.4 ≤ F < 0.8 µm
F < 0.4 µm
Alanine
Citric acid
a
a
1000
200
ab
abc
a
ab
ab
ab
ab
ab
ab
ab
bc
bc
bc
bc
900
150
cd
de
 C-CO2 (µg g-1 dry soil h-1)
 C-CO2 (µg g-1 dry soil h-1)
800
100
b
b
b
e
e
e
700
50
RW4
H2O2
F ≥ 3 µm
Heat shock
Gentamicin
Ramoplanin
NT control
Original soil
Ciprofloxacin
0.8 ≤ F < 0.3 µm
0.4 ≤ F < 0.8 µm
F < 0.4 µm
RW4
H2O2
F ≥ 3 µm
Heat shock
Gentamicin
Ramoplanin
NT control
Original soil
Ciprofloxacin
0.8 ≤ F < 0.3 µm
0.4 ≤ F < 0.8 µm
F < 0.4 µm
600
0
Malic acid
Arabinose
a
a
1000
200
a
ab
a
ab
ab
ab
ab
ab
ab
ab
ab
800
150
b
bc
bc
bc
bc
cd
 C-CO2 (µg g-1 dry soil h-1)
 C-CO2 (µg g-1 dry soil h-1)
600
100
de
ef
f
ef
b
400
50
RW4
H2O2
F ≥ 3 µm
Heat shock
Gentamicin
Ramoplanin
NT control
Original soil
Ciprofloxacin
0.8 ≤ F < 0.3 µm
0.4 ≤ F < 0.8 µm
F < 0.4 µm
RW4
H2O2
F ≥ 3 µm
Heat shock
Gentamicin
Ramoplanin
NT control
Original soil
Ciprofloxacin
0.8 ≤ F < 0.3 µm
0.4 ≤ F < 0.8 µm
F < 0.4 µm
200
0
Treatments
Treatments
Supplementary Fig. 11 a Substrate-induced respiration as measured by the MicroResp™ method using the substrates galactose, arginine, citric acid, alanine, malic acid and arabinose (mean ± s.e.). Different letters above the bars indicate significant differences according to Tukey’s test (p-value < 0.05).

## Slide 13
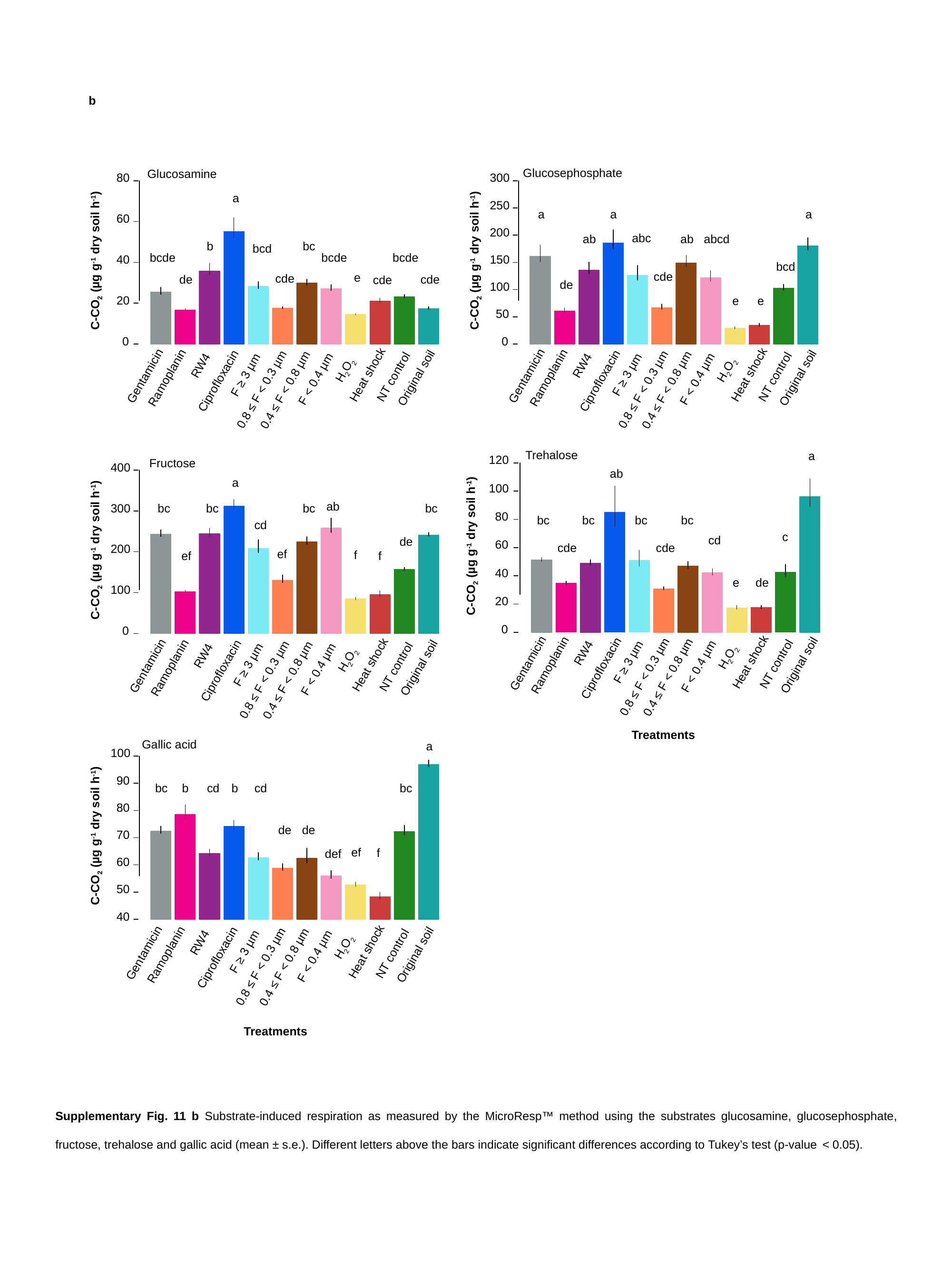

b
Glucosamine
Glucosephosphate
80
300
a
a
a
a
250
60
abc
ab
ab
abcd
200
b
bc
bcd
bcde
bcde
bcde
bcd
 C-CO2 (µg g-1 dry soil h-1)
 C-CO2 (µg g-1 dry soil h-1)
40
150
cde
e
cde
cde
de
cde
de
100
e
e
20
50
RW4
H2O2
F ≥ 3 µm
Heat shock
Gentamicin
Ramoplanin
NT control
Original soil
Ciprofloxacin
0.8 ≤ F < 0.3 µm
0.4 ≤ F < 0.8 µm
F < 0.4 µm
RW4
H2O2
F ≥ 3 µm
Heat shock
Gentamicin
Ramoplanin
NT control
Original soil
Ciprofloxacin
0.8 ≤ F < 0.3 µm
0.4 ≤ F < 0.8 µm
F < 0.4 µm
0
0
a
Trehalose
Fructose
120
ab
400
a
100
ab
bc
bc
bc
bc
300
bc
bc
bc
bc
cd
80
c
cd
de
cde
cde
 C-CO2 (µg g-1 dry soil h-1)
ef
f
60
ef
f
 C-CO2 (µg g-1 dry soil h-1)
200
de
e
40
100
20
RW4
H2O2
F ≥ 3 µm
Heat shock
Gentamicin
Ramoplanin
NT control
Original soil
Ciprofloxacin
0.8 ≤ F < 0.3 µm
0.4 ≤ F < 0.8 µm
F < 0.4 µm
RW4
H2O2
F ≥ 3 µm
Heat shock
Gentamicin
Ramoplanin
NT control
Original soil
Ciprofloxacin
0.8 ≤ F < 0.3 µm
0.4 ≤ F < 0.8 µm
F < 0.4 µm
0
0
Treatments
a
Gallic acid
100
bc
b
cd
b
cd
bc
90
80
de
de
 C-CO2 (µg g-1 dry soil h-1)
70
ef
f
def
60
50
RW4
H2O2
F ≥ 3 µm
Heat shock
Gentamicin
Ramoplanin
NT control
Original soil
Ciprofloxacin
0.8 ≤ F < 0.3 µm
0.4 ≤ F < 0.8 µm
F < 0.4 µm
40
Treatments
Supplementary Fig. 11 b Substrate-induced respiration as measured by the MicroResp™ method using the substrates glucosamine, glucosephosphate, fructose, trehalose and gallic acid (mean ± s.e.). Different letters above the bars indicate significant differences according to Tukey’s test (p-value < 0.05).
